# A Synopsis of *Orthotrichum* s. lato (Bryophyta, Orthotrichaceae) in China, with Distribution Maps and a Key to Determination

**DOI:** 10.3390/plants10030499

**Published:** 2021-03-08

**Authors:** Vítězslav Plášek, Zuzana Komínková, Ryszard Ochyra, Lucie Fialová, Shuiliang Guo, Mamtimin Sulayman

**Affiliations:** 1 Department of Botany, University of Ostrava, Chittussiho 10, 710 00 Ostrava, Czech Republic; zuzana.skoupa@email.cz (Z.K.); fialova.lucie92@gmail.com (L.F.); 2 Laboratory of Bryology, Władysław Szafer Institute of Botany, Polish Academy of Sciences, Lubicz 46, 31-512 Kraków, Poland; r.ochyra@botany.pl; 3 College of Life and Environmental Sciences, Shanghai Normal University, 100 Guilin Road, Shanghai 200234, China; gsg@shnu.edu.cn; 4 College of Life Science and Technology of Xinjiang University, Urumqi 830046, China; mamtimin@xju.edu.cn

**Keywords:** Asia, bryophytes, distribution, endemics, floristic elements, Musci, Orthotrichaceae, phytogeography

## Abstract

A total of 46 species and two varieties of the traditionally interpreted genus *Orthotrichum* are currently known to occur in China. They represent five genera, including *Orthotrichum* (29 species), *Lewinskya* (14 species and two varieties), and *Nyholmiella* and *Leratia* that are represented by a single species each. The fifth genus *Florschuetziella*, also consisting of only one species, *F. scaberrima*, is an entirely neglected representative of the China’s moss flora. A list of all accepted taxa is presented and for each taxon all literature records and herbarium specimens are enumerated for provinces in which they have been recorded, and their distribution is mapped. A key to determination of Chinese orthotrichalean mosses is presented. A chronological list of 63 species and varieties and two designations, *O. catagonioides* and *O. microsporum* which have never been validly published, reported from China in the years 1892–2020 is presented. Four species, *Orthotrichum brasii*, *O. hooglandii*, *O. elegans* and *O. gymnostomum* are excluded from the bryoflora of China and *Lewinskya affinis* var. *bohemica* and *Orthotrichum schimperi* are recorded for the first time from this country. Phytogeography of the Chinese taxa of the orthotrichalean mosses is considered and they are grouped into eight phytogeographical elements and five sub-elements.

## 1. Introduction

The traditionally conceived genus *Orthotrichum* Hedw. is one of the largest moss genera in the bryoflora of China. In the Chinese version of the moss Flora of this country 28 species and one variety are recorded [1], whereas in the English version of this work this number is increased to 32 species and one variety [2]. Two years later, in the catalogue of Chinese bryophyte species, Jia and He [3] included five additional species in *Orthotrichum* and in this way the genus reached 36 species and one variety. It is because these authors placed *O. exiguum* Sull. in the separate genus *Leratia* Broth. & Paris. *Orthotrichum* thus became the fifth largest moss genus in China after *Fissidens* Hedw. (56 species), *Brachythecium* Schimp. (50 species), and *Sphagnum* L. and *Bryum* Hedw. (each with 47 species). Finally, in the newest taxonomic revision of *Orthotrichum* only 26 species and two varieties have been accepted as occurring in China, whereas two species have been excluded and seven recognised as of doubtful occurrence in the moss flora of this country [4].

In all aforementioned taxonomic treatments the traditional concept of *Orthotrichum* was adopted. However, as is the case with most of the larger moss genera, *Orthotrichum* is a heterogeneous taxon. As a result of taxonomic and molecular studies which have confirmed its polyphyly, three segregates have been split from it, including *Nyholmiella* Holmen & E. Warncke, *Pulvigera* Plášek, Sawicki & Ochyra and *Lewinskya* F. Lara, Garilleti & Goffinet [5,6,7,8,9,10]. In addition, *O. exiguum*, a highly isolated species in *Orthotrichum* which was usually placed in a separate subgenus, *O.* subg. *Exiguifolium* Vitt, has been transferred to the genus *Leratia* as *L. exigua* (Sull.) Goffinet [11].

Putting aside different generic placements of species belonging to the traditionally interpreted genus Orthotrichum, the existence of very large differences in the number of accepted orthotrichalean species in the moss flora of China must raise questions about the reasons for these discrepancies, especially because they concern outstanding species, the distinctiveness of which is indisputable and widely accepted. The main aim of the present work is an attempt to resolve these controversial issues and to give a clear and lucid answer how many species of orthotrichalean mosses are actually known to occur in China. A critical revision of all available voucher specimens has been undertaken. A considerable amount of literature data, often published in Chinese, as well as a large number of herbarium specimens housed in Chinese, European and North American herbaria, have been critically evaluated, resulting in a new catalogue and an updated key to identification of orthotrichalean mosses in China.

## 2. A Brief Survey of Studies on Orthotrichalean Mosses in China

It is commonly believed [12,13,14] that the first species of the traditionally conceived genus *Orthotrichum* were reported from China by Mitten [15] in his *Musci Indiae Orientalis*. He recorded *Orthotrichum anomalum* Hedw., *O. crenulatum* Mitt., and *O. speciosum* Nees from “Tibet occidentalis” from specimens collected by Thomas Thomson (1817–1878). Unfortunately, this is not entirely correct because Thomson’s specimens cited by Mitten [15] were in fact provisionally named by W. Wilson and published already two years earlier by Mitten and Wilson [16]. Moreover, they were not collected in Chinese Tibet (Xizang) but in Kashmir. Thomas Thomson was a British surgeon who joined the East India Company and in 1840 was appointed Assistant Surgeon in the Bengal Army, serving in various campaigns in Afghanistan and India. During 1847–1848 he was, together with Lieutnant-Colonel Sir Henry Strachey (1816–1912), a commissioner of the survey of the boundary between Kashmir and Tibet led by Major General Sir Alexander Cunningham (1814–1893), which explored the northern frontier of Kashmir, along the Karakoram Range [17].

Thomas Thomson collected two specimens of orthotrichalean mosses from “Tibet occid. temp.”, i.e., from the temperate region in western Tibet. One of these, designated as No. 209, was gathered at an altitude of 11,000 ft (3355 m) at the Nubra River valley in the Indian union territory of Ladakh. William Wilson determined it, with a question mark, as *Orthotrichum leptocarpum* Bruch & Schimp. [16] and the moss was subsequently redetermined by Mitten [15] as *O. anomalum*. The second specimen, bearing the collecting number No. 251, was collected by Thomson at Rondu at an elevation of 6000 ft (=1830 m) in the temperate region of western Tibet, which at present lies in the Rondu (or Roundu) District of the Pakistan-administrated mountainous region of Gilgit-Baltistan. The specimen was initially determined by W. Wilson as an unnamed variety of *O. anomalum*, although he suggested that possibly it represented a new undescribed species [16]. Mitten [15] recognised it as a species new to sciences, *O. crenulatum*. Interestingly, Mitten [15] placed the specimen No. 251 among specimens examined of *O. anomalum* and left the type specimen of *O. crenulatum* without any collecting number, only with a note that the moss was found “inter caespites *Drummondiae Thomsoni*” which was collected at the same locality as No. 251. Thus, the localities of the two specimens of *Orthotrichum* collected by Thomson in western Tibet are actually not situated in China, but in Pakistan and India.

Mitten [15] recorded the third species of *Orthotrichum* from the alpine region in western Tibet, namely *O. speciosum*, represented by the specimen No. 211 collected by Th. Thomson but without details of the locality. This is evidently an erroneous ascription of this specimen to this collector because earlier Mitten and Wilson [16] had clearly stated that the specimen No. 211 was gathered by Henry Strachey on the top of Hera La mountain at an altitude of 18,700 ft (5703 m). In addition, H. Strachey also collected at this station the specimen No. 193 which was named by W. Wilson, with a question mark, as *Orthotrichum leptocarpum* [16]. It was not cited by Mitten [15] but the other specimen No. 209 so determined by W. Wilson was considered *O. anomalum* by Mitten [15]. Hera La pass is situated in the Indian union territory of Ladakh north of Leh City, approximately midway between the Nubra River and the upper course of the Shoyk River at an elevation of about 4600 m [18]. The road Ke Chu runs through this pass from the Ugu village in the Nubra River valley via Kela Tso lake to the Tangtse village in the Shoyk River valley. The pass crosses a mountain ridge rising on its northern side to a height of 5720 m, on the summit of which at lat. 34°02′27.37″N and long. 78°01′48.50″E Henry Strachey collected several specimens of moss, including the mentioned *Orthotrichum* species.

It is worth mentioning that British explorers did visit the Chinese part of Tibet but, unfortunately, they made no collections of bryophytes. In 1846 Henry Strachey made an unauthorised visit to the Tibetan region surrounding Lakes Manasarowar (*Tibetan* Mapam Yumco) and Rakshatal (*Tibetan* La’nga Co) [19,20]. This journey was repeated by his brother Richard Strachey in the company of the botanist J. E. Winterbottom in 1848 [21].

The discoveries of orthotrichalean mosses in China are closely related to the history of bryological exploration of this vast Asian country, as outlined by Koponen [22]. The taxa of the traditionally interpreted genus *Orthotrichum* which have been recorded from China are presented in chronological order in Table 1. The long-lasting Chinese policy of isolating the country from the rest of the world meant that until the middle of the nineteenth century there were practically no collections of bryophytes from China. The only exceptions were some moss records from the eastern coastal regions [23,24,25,26,27,28,29]. After the end of the second “Opium War” (1856–1860), the situation improved markedly and as a result of the peace treaty, signed in 1861, visitors could travel to the interior of the country and missionaries were allowed to work wherever they wanted. This had far-reaching consequences for botanical and bryological research in China.

The first major collections of mosses in China were made by French missionary Jean Marie Delavay (1838–1895) who worked from 1881 until his death in northwest Yunnan, and Italian missionary Giuseppe Giraldi (?–1901) who collected plants in Shaanxi Province in the years 1890–1895. The moss collection of the former was studied by Bescherelle [30,31], who reported *Lewinskya hookeri* (as *Orthotrichum hookeri*), and after his death one of his specimens was recognised as a new species, *Racomitrium delavayi*, by V. F. Brotherus and Paris [32], which actually proved to be *O. callistomum* [12]. Müller [33,34,35] studied the moss specimens collected by Giraldi and described no fewer than four new species of *Orthotrichum* which are at present accepted as distinct taxa. After Müller’s death in 1899, Giraldi’s material continued to be examined by V. F. Brotherus and the results were published by Levier [36].

**Table 1:** A list in chronological order of species of the *sensu lato* genus *Orthotrichum* described and recorded from China between 1857 and 2020. Names in capital letters refer to currently accepted taxa; names in boldface refer to taxa described from the Chinese material; names in capital letters and boldface refer to taxa described from China and accepted in modern taxonomy; underlined names refer to currently accepted species and indicate their first report from China under this name, though sometimes in a different genus.

No.	Species	Year, Publication	Current Status	First Synonymisation or Change of the Taxonomic Status
1.	*Orthotrichum hookeri* Wilson *ex* Mitt.	1892 [31]	*LEWINSKYA HOOKERI* (Wilson *ex* Mitt.) F.Lara, Garilleti & Goffinet	
2.	***Orthotrichum leiolecythis*** Müll.Hal.	1896 [33]	*LEWINSKYA LEIOLECYTHIS* (Müll.Hal.) F.Lara, Garilleti & Goffinet	
3.	***ORTHOTRICHUM ERUBESCENS*** Müll.Hal.	1897 [34]		
4.	***ORTHOTRICHUM REVOLUTUM*** Müll.Hal.	1897 [34]		
5.	***Orthotrichum macrosporum*** Müll.Hal.	1898 [35]	*LEWINSKYA HOOKERI* var. *GRANULATA *(Lewinsky) F.Lara, Garilleti & Goffinet	[12]
6.	***Racomitrium delavayi*** Broth. & Paris	1908 [32]	*ORTHOTRICHUM CALLISTOMUM *Fisch.-Oost. *ex* Bruch & Schimp.	[12]
7.	***Orthotrichum microsporum*** Müll.Hal. *in* Levier, *nom. nud*.	1906 [36]	*Lewinskya hookeri* var. *granulata* (Lewinsky) F.Lara, Garilleti & Goffinet	[12]
8.	*Orthotrichum rupestre* Schwägr.	1906 [36]	*LEWINSKYA RUPESTRIS* (Schwägr.) F.Lara, Garilleti & Goffinet	
9.	***Orthotrichum fortunatii*** Thér.	1909 [37]	*Orthotrichum erubescens* Müll.Hal.	[12]
10.	***Orthotrichum decurrens*** Thér.	1909 [37]	*LERATIA EXIGUA *(Sull.) Goffinet	[38]
11.	***Orthotrichum courtouisii*** Broth. & Paris	1910 [39]	*ORTHOTRICHUM CONSOBRINUM* Cardot	[12,40]
12.	***Orthotrichum callistomoides*** Broth.	1924 [41]	*Orthotrichum callistomum*Fisch.-Oost. *ex* Bruch & Schimp.	[12]
13.	***ORTHOTRICHUM CRISPIFOLIUM*** Broth.	1929 [42]		
14.	***Orthotrichum scaberrimum*** Broth.	1929 [42]	*FLORSCHUETZIELLA SCABERRIMA *(Broth.) Vitt	[43]
15.	*ORTHOTRICHUM URNIGERUM*Myrin	1937 [44]		
16.	***Orthotrichum szuchuanicum*** P.C.Chen	1943 [45]	*Leratia exigua* (Sull.) Goffinet	[12]
17.	*Orthotrichum leiocarpum* Bruch & Schimp., *nom. illeg.*	1963 [46]	*LEWINSKYA STRIATA* (Hedw.) F.Lara, Garilleti & Goffinet	
18.	*Orthotrichum affine* Brid.	1977 [47]	*LEWINSKYA AFFINIS* (Brid.) F.Lara, Garilleti & Goffinet	
19.	*Orthotrichum speciosum* Ness	1977 [47]	*LEWINSKYA SPECIOSA* (Nees) F.Lara, Garilleti & Goffinet	
20.	*Orthotrichum striatum* Hedw.	1977 [47]	*Lewinskya striata* (Hedw.) F.Lara, Garilleti & Goffinet	
21.	*Orthotrichum obtusifolium* Brid.	1977 [47]	*NYHOLMIELLA OBTUSIFOLIA*(Brid.) Holmen & E.Warncke	
22.	*ORTHOTRICHUM ANOMALUM* Hedw.	1978 [48]		
23.	*Orthotrichum exiguum* Sull.	1978 [49]	*Leratia exigua* (Sull.) Goffinet	
24.	***ORTHOTRICHUM CATAGONIOIDES*** Broth. *in* P.C.Chen, *nom. nud*.	1978 [49]		
25.	*Orthotrichum macounii* Austin var. *japonicum* Z.Iwats.	1985 [50]	*LEWINSKYA IWATSUKII *Ignatov	[51]
26.	***Orthotrichum pulchrum*** Lewinsky	1992 [12]	*LEWINSKYA PULCHRA *(Lewinsky) F.Lara, Garilleti & Goffinet	
27.	***Orthotrichum taiwanense*** Lewinsky	1992 [12]	*LEWINSKYA TAIWANENSIS *(Lewinsky) F.Lara, Garilleti & Goffinet	
28.	***Orthotrichum dasymitrium*** Lewinsky	1992 [52]	*LEWINSKYA DASYMITRIA *(Lewinsky) F.Lara, Garilleti and Goffinet	
29.	***Orthotrichum erosum*** Lewinsky	1992 [12]	*LEWINSKYA EROSA *(Lewinsky) F.Lara, Garilleti & Goffinet	
30.	*Orthotrichum laevigatum* Zett. var. *japonicum* (Z.Iwats.) Lewinsky	1992 [12]	*Lewinskya iwatsukii* Ignatov	[51]
31.	*Orthotrichum sordidum* Sull. & Lesq.	1992 [12]	*LEWINSKYA SORDIDA *(Sull. and Lesq.) F.Lara, Garilleti & Goffinet	
32.	*Orthotrichum exiguum* Sull.	1992 [12]	*Leratia exigua* (Sull.) Goffinet	
33.	*ORTHOTRICHUM GRIFFITHII* Mitt. *ex* Dixon	1992 [12]		
34.	***ORTHOTRICHUM SUBPUMILUM*** E.B.Bartram *ex* Lewinsky	1992 [12]		
35.	*ORTHOTRICHUM PUMILUM*Sw.	1992 [12]		
36.	***ORTHOTRICHUM SINUOSUM*** Lewinsky	1992 [12]		
37.	*Orthotrichum consobrinum* Cardot	1992 [12]		
38.	*Orthotrichum callistomum *Fisch.-Oost. *ex* Bruch & Schimp.	1992 [12]		
39.	*ORTHOTRICHUM CUPULATUM* Brid.	1995 [53]		
40.	*ORTHOTRICHUM PALLENS* Brid.	1995 [53]		
41.	*Orthotrichum speciosum* Nees var. *elegans* Hook. & Grev.	1995 [53]	*LEWINSKYA ELEGANS* (Hook. amnd Grev.) F.Lara, Garilleti & Goffinet—excluded	
42.	*ORTHOTRICHUM HALLII* Sull. & Lesq.	1995 [54]		
43.	***ORTHOTRICHUM NOTABILE*** Lewinsky	1995 [55]		
44.	*ORTHOTRICHUM PELLUCIDUM* Lindb.	1995 [54]		
45.	*ORTHOTRICHUM STRAMINEUM* Brid.	1996 [56]		
46.	***Orthomitrium tuberculatum*** Lewinsky & Crosby	1996 [57]	*ORTHOTRICHUM JETTEAE* B.H.Allen	[58]
47.	***Orthomitrium schofieldii*** B.C.Tan & Y.Jia	1997 [57]	*ORTHOTRICHUM SCHOFIELDII* (B.C.Tan & Y.Jia) B.H.Allen	[58]
48.	*ORTHOTRICHUM CRENULATUM* Mitt.	1999 [59]		
49.	***ORTHOTRICHUM LAXUM***Lewinsky	1999 [60]		
50.	***ORTHOTRICHUM VERMIFERUM*** Lewinsky	1999 [61]		
51.	*Orthotrichum gymnostomum* Brid.	2003 [62]	*NYHOLMIELLA GYMNOSTOMA *(Brid.) Holmen & E.Warncke—Excluded	
52.	*ORTHOTRICHUM HOOGLANDII* E.B.Bartram	2011 [1]	Excluded	
53.	*Orthotrichum brasii* E.B.Bartram	2011 [1]	*LEWINSKYA BRASSII* (E.B.Bartram) F.Lara, Garilleti & Goffinet—Excluded	
54.	*ORTHOTRICHUM IBUKIENSE* Toyama	2011 [1]		
55.	*ORTHOTRICHUM PAMIRICUM* Plášek & Sawicki	2016 [63]		
56.	*LEWINSKYA VLADIKAVKANA*(Venturi) F.Lara, Garilleti & Goffinet	2017 [64]		
57.	*ORTHOTRICHUM ALPESTRE* Wilson	2017 [64]		
58.	*ORTHOTRICHUM MORAVICUM* Plášek & Sawicki	2018 [65]		
59.	*ORTHOTRICHUM ROGERI* Brid.	2018 [65]		
60.	*ORTHOTRICHUM SCANICUM* Grönvall	2018 [65]		
61.	*LEWINSKYA GRAPHIOMITRIA* (Müll. Hal. ex Beckett) F. Lara, Garilleti & Goffinet	2020 [66]		
62.	*LEWINSKYA AFFINIS* var. *BOHEMICA* (Plášek & Sawicki) Plášek	This article		
63.	*ORTHOTRICHUM SCHIMPERI* Hammar	This article		

Surprisingly, all the early moss collections from China were made by non-botanists, with the only exception being the Austrian botanist Heinrich Freiherr von Handel-Mazzetti (1882–1940). In the years 1914–1918 he botanised in Yunnan and Sichuan and some adjoining provinces and made the richest moss collection ever brought back from China, consisting of 1484 specimens [67]. The collection was studied by Brotherus [41,42,68], who determined 612 species, of which 232 species, 28 varieties and 3 forms were new to science, including three new species of *Orthotrichum* [69].

In total, by the end of World War II, 17 species of mosses of the genus *Orthotrichum* had been found in China, 12 of which were described as new to science, and the name of one not validly published. This first period of study on this genus in China ends with the description of a new species, *O. szuchuanicum*, by Chen [45], the first and until then the only species of this genus described by a Chinese bryologist.

After World War II local students, centred around Pan-Chieh Chen (1907–1970), the founder of Chinesae modern bryology [70], actively participated in the study of the bryoflora of China. The results of these studies were published in several local Floras, in which also *Orthotrichum* species were described and illustrated. The earliest regional moss Flora was completed by Gao [47] who reported four species of *Orthotrichum* from Northeast China, including *O. affine*, *O. obtusifolium*, *O. speciosum*, and *O. striatum*. A year later, Zhang [48] published a volume devoted to the mosses in *Flora Tsinglingensis* and recorded *O. anomalum* from the Qinling Mountains in Shaanxi Province. In a Flora of the mosses of Xizang [71], the Orthotrichaceae were contributed by Hu and Wang [50] who reported *O. anomalum* and *O. macounii* var. *japonicum* from this autonomous region. Lin [72] recorded *O. callistomum* from Taiwan, the first record of this genus from this insular province. In *Cryptogamic Flora of the Yangtze Delta and adjacent regions* Liu [73] provided records of two *Orthotrichum* species, *O. consobrinum* in Jiangsu and *O. courtoisii* in Jiangsu and Shanghai, and Aur et al. [74] reported *O. affine* from Heilongjiang Province.

Additionally, numerous Chinese bryologists have investigated bryophyte species in nature reserves or other geographical regions and published papers including information on *Orthotrichum* species. For example, Bai [75] reported *O. affine* from Inner Mongolia and Wu [76] recorded *O. leiolecythis* from Xishuangbanna in Yunnan. Recently, several valuable treatments on the mosses of Guizhou Province have been published [77,78,79,80] and moss Floras of Inner Mongolia [81] and the Helan Mountains on the border of Inner Mongolia and Ningxia Province [82] are available. In all these works species of *Orthotrichum* are described and illustrated. Orthotrichalean species have also been considered in some iconographic works of Chinese mosses including excellent photographic atlases [62,83,84].

Apart from these local Floras and checklists of mosses for some provinces, for example Xinjiang [53], Gansu [85] and Zhejiang [86], in the last fifty years a number of taxonomic studies covering the entire country and in which also species of *Orthotrichum* have been considered, have been published in China. The first such work was *Genera muscorum sinicorum* [49,87], a synopsis of all species, genera and supragenetic taxa known in this country. In the mid-1980s the first checklist of Chinese mosses was published [88] and its second, updated edition appeared a decade later [13].

Lewinsky [12] published a taxonomic revision of the genus *Orthotrichum* in Southeast Asia, in which 24 species of *Orthotrichum* are included from China. Five of these, *O. pulchrum* Lewinsky, *O. taiwanense* Lewinsky, *O. erosum* Lewinsky, *O. subpumilum* E.B.Bartram *ex* Lewinsky and *O. sinuosum* Lewinsky, were described as new to science from Chinese specimens. Subsequently, three additional new species of *Orthotrichum* from China were described, including *O. notabile* Lewinsky from Hongyuan County in Sichuan Province [55], *O. laxum* Lewinsky from Maqin in Qinghai [61] and *O. vermiferum* Lewinsky from Huzhu in Qinghai [60]. Additionally, Lewinsky-Haapasaari and Crosby [89] described a new monotypic genus, *Orthomitrium* Lewinsky & Crosby, with a single species, *O. tuberculatum* Lewinsky & Crosby from Guizhou Province, and a year later Tan & Jia [57] added another new species to this genus, *O. schofieldii* B.C. Tan and Y. Jia, from Qinghai and Sichuan Provinces. However, Allen [58] considered *Orthomitrium* to be congeneric with *Orthotrichum* and, accordingly, these two species were transferred to the latter genus as *O. jetteae* B.H.Allen and *O. schofieldii* (Lewinsky & Crosby) B.H.Allen. After publication of these taxonomic novelties, Mo et al. [90] briefly reviewed the taxonomical researches on Chinese Orthotrichaceae and Hu et al. [91] constructed a phylogenetic tree of the family Orthotrichaceae based on three gene sequences including chloroplast *rbc*L, tRNA-Leu (*trn*L) gene, and NADH dehydrogenase subunit 5 gene, the tree including eight species of *Orthotrichum*.

In the two decades at the turn of the twentieth and the present twenty first centuries two major bryological projects intended to complete the descriptive Floras of mosses of China have successfully been accomplished. In the years 1994–2011, eight volumes of the Chinese version of *Flora bryophytorum sinicorum* were published and the fifth volume included the treatment of the Orthotrichaceae [1] and in the years 1999–2011, its analogical, though not entirely identical, English version was completed, with the treatise of the Orthotrichaceae in the fifth volume [2]. After publication of these two parallel taxonomic treatments of *Orthotrichum* in China, in which different numbers of species were accepted between the Chinese and English versions, namely 28 and 32, respectively, the third monograph of this genus was published in which only 26 species were considered to occur in China [14]. 2. Material and Methods

The authors have intended to study any available publications dealing with taxonomy and distribution of orthotrichalean mosses in China, including many local bryological works published in the Chinese language. During the course of the present study, the herbarium collections from the following Chinese and European herbaria have been revised (acronyms of the herbaria according to *Index herbariorum* but for some Chinese herbaria they are unauthorised and introduced only for the purposes of the present article; they are distinguished by not using bold letters):

**C**—University of Copenhagen, Denmark;
**E**—Royal Botanic Garden Edinburgh, Scotland, U.K;
GuZU—Guizhou University, China;
**H**—University of Helsinky, Finland;
**HSNU**—Herbaria of East China Normal University, China;
HuBU—Hubei University, China;
**IFP**—Institute of Applied Ecology, Academia Sinica, Shenyang, China;
InMU—Inner Mongolia University, China;
**KRAM**—Herbarium of the W. Szafer Institute of Botany, Polish Academy of Sciences, Krakow, Poland;
**KUN**—Herbarium of Kunming Institute of Botany, the Chinese Academy of Sciences, China;
**OSTR**—University of Ostrava, Czech Republic;
**PC**—Muséum National d’Historie Naturelle, Paris, France;
**PE**—Institut of Botany, Chinese Academy of Sience, Beijing, China;
ShM—Shanghai Museum, China;
ShU—Shanghai Normal University, China;
**W**—Naturhistorisches Museum, Wien, Austria;
**XJU**—Xinjiang University, China.

**Figure 1:**
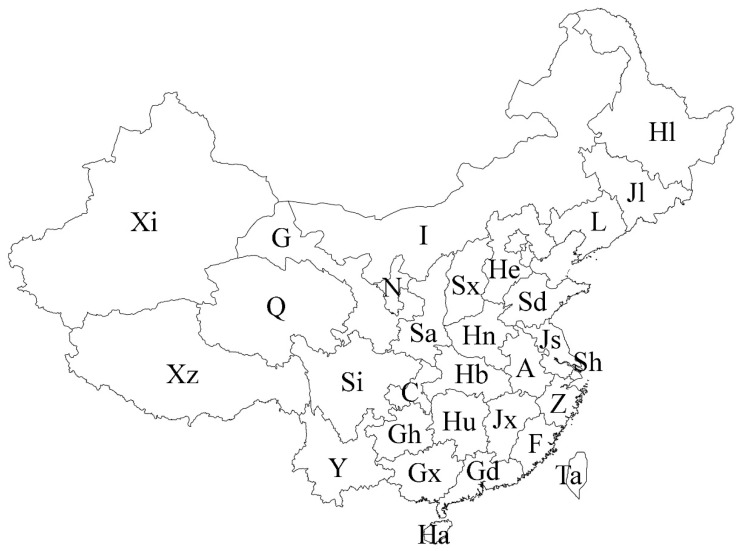
Provinces and autonomous regions of China. **A**—Anhui, **C**—Chongqing, **F**—Fujian, **G**—Gansu, **Gd**—Guangdong, **Gh**—Guizhou, **Gx**—Guangxi, **Ha**—Hainan, **Hb**—Hubei, **He**—Hebei, **Hl**—Heilongjiang, **Hn**—Henan, **Hu**—Hunan, **I**—Inner Mongolia, **Jl**—Jilin, **Js**—Jiangsu, **Jx**—Jiangxi, **L**—Liaoning, **N**—Ningxia, **Q**—Qinghai, **Sa**—Shaanxi, **Sd**—Shandong, **Sh**—Shanghai, **Si**—Sichuan, **Sx**—Shanxi, **Ta**—Taiwan, **Xi**—Xinjiang, **Xz**—Xizang, **Y**—Yunnan, **Z**—Zhejiang.

Since 2015, the Czech–Polish–Chinese cooperation has focused on a critical revision of the genus *Orthotrichum s.lat.* in China. After extraction of literature data and a revision of orthotrichaceous moss specimens housed in the major European and Chinese herbaria, field researches in twelve provinces of China were carried out, including Anhui, Guizhou, Henan, Hubei, Jiangsu, Shaanxi, Shanxi, Shanghai, Sichuan, Xinjiang, Yunnan, Zhejiang (Figure 1).

The GPS coordinates follow the WGS84 system. Nomenclature follows Lara et al. [10] and Procházková and Plášek [92]. Duplicates of herbarium specimens and own materials collected during the field research are deposited in OSTR and, partly, in KRAM.

## 3. Results

As a result of a critical revision of all available data, occurrence of 46 species and two varieties of the orthotrichalean mosses is confirmed for China (Table 2). The recent study of the authors yielded nine species new to China, including *Lewinskya affinis* var. *bohemica*, *L. graphiomitria*, *L. vladikavkana*, *Orthotrichum alpestre*, *O. moravicum*, *O. pamiricum*, *O. rogeri*, *O. scanicum*, and *O. schimperi* [63,64,65,66], of which the first and the last taxon are newly recorded from China in the present paper. In addition, another 36 species have been recorded for the first time from various provinces of China [63,64,65,66,93,94,95,96,97,98,99].

**Figure 2:**
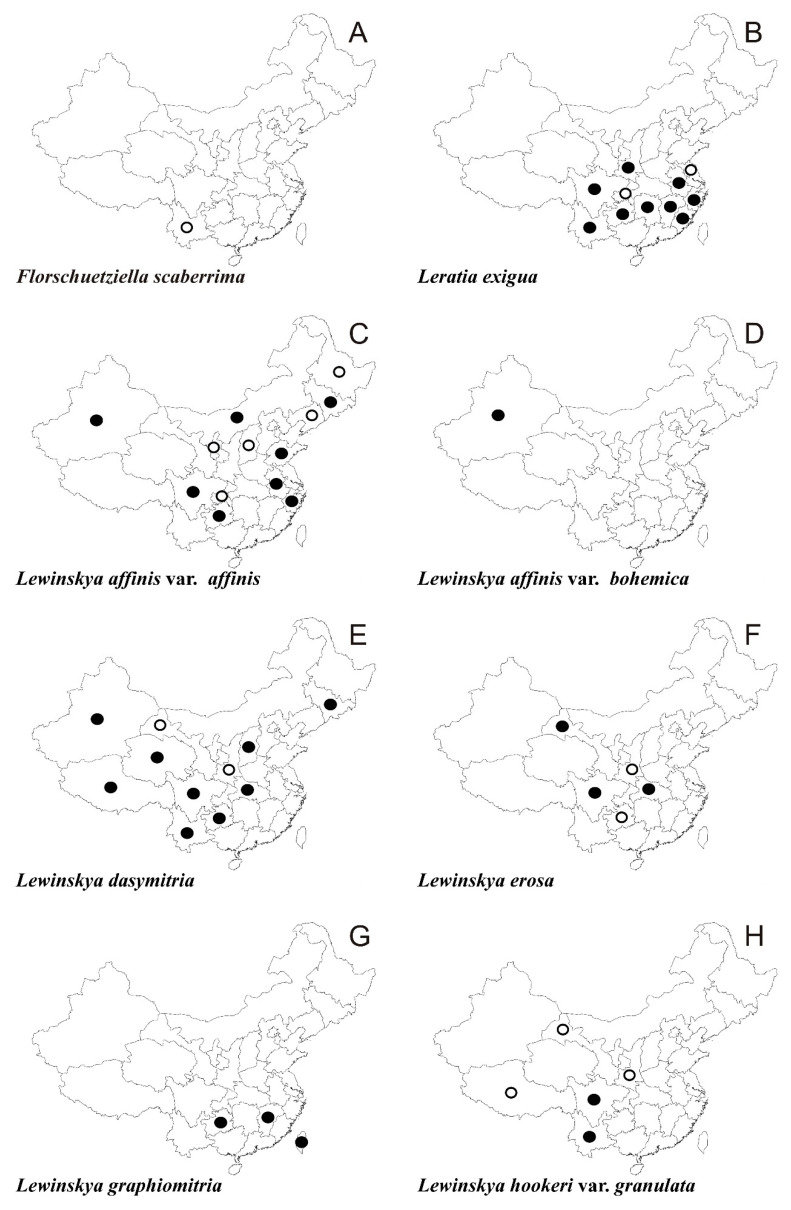
Distribution maps for taxa of the genera *Florschuetziella*, *Leratia* and *Lewinskya* in China’s provinces.

**Figure 3:**
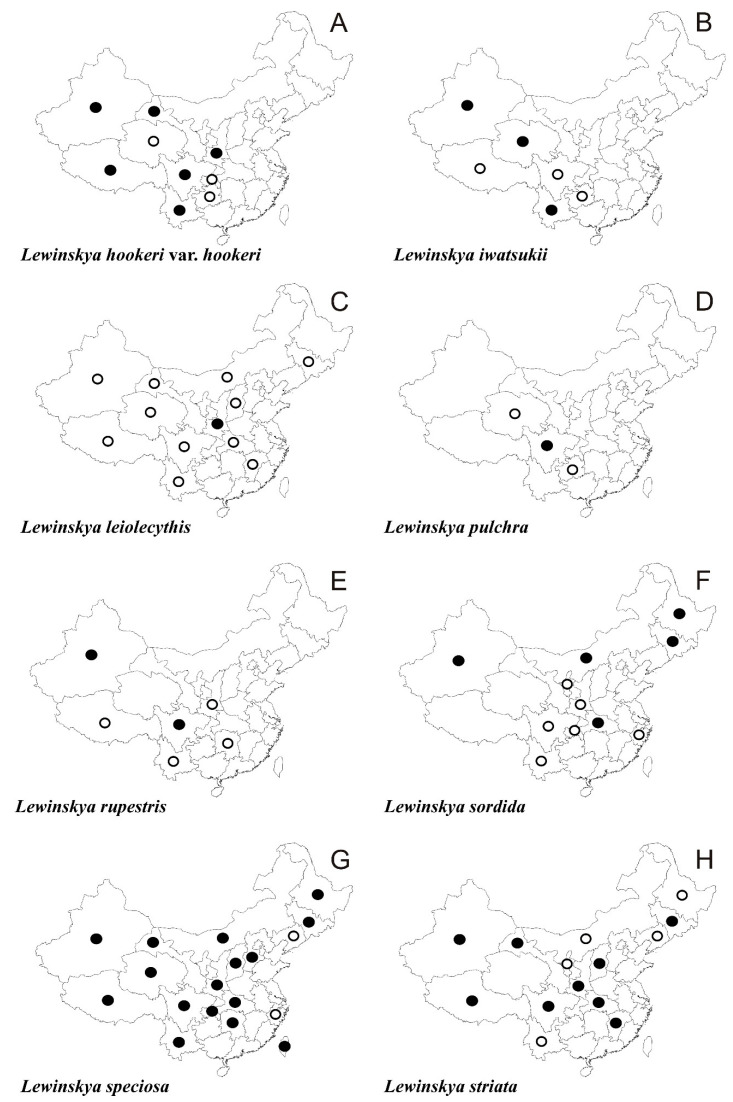
Distribution map for taxa of the genus *Lewinskya* in China’s provinces.

**Figure 4:**
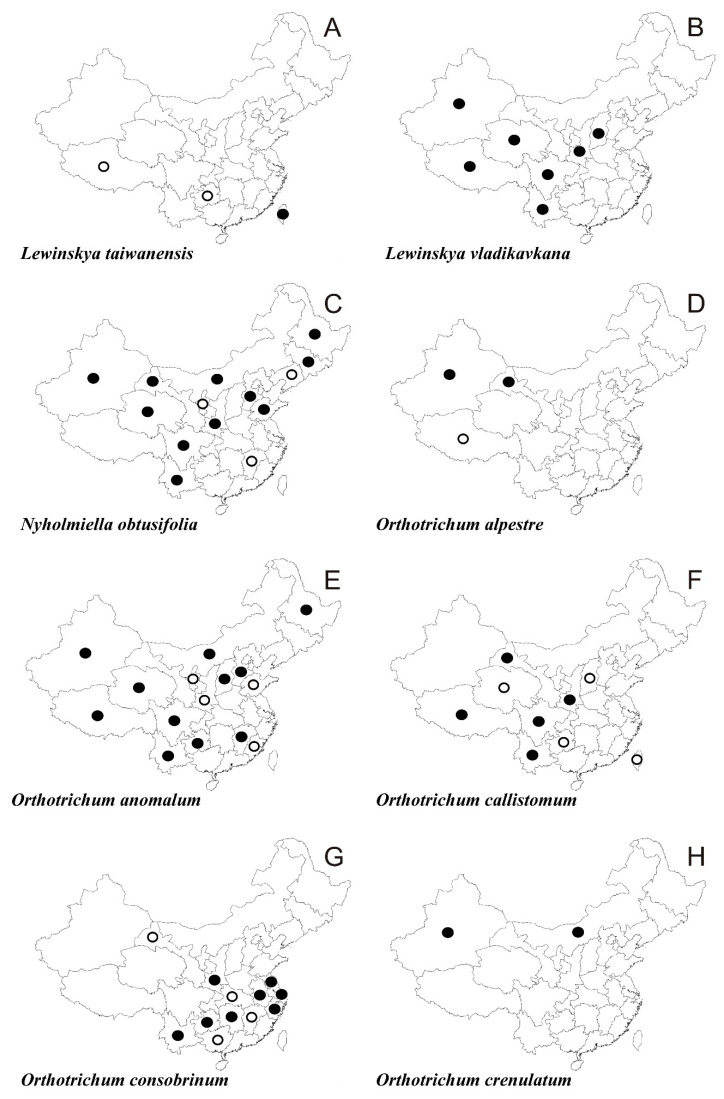
Distribution maps for species of the genera *Lewinskya*, *Nyholmiella,* and *Orthotrichum* in China’s provinces.

**Figure 5:**
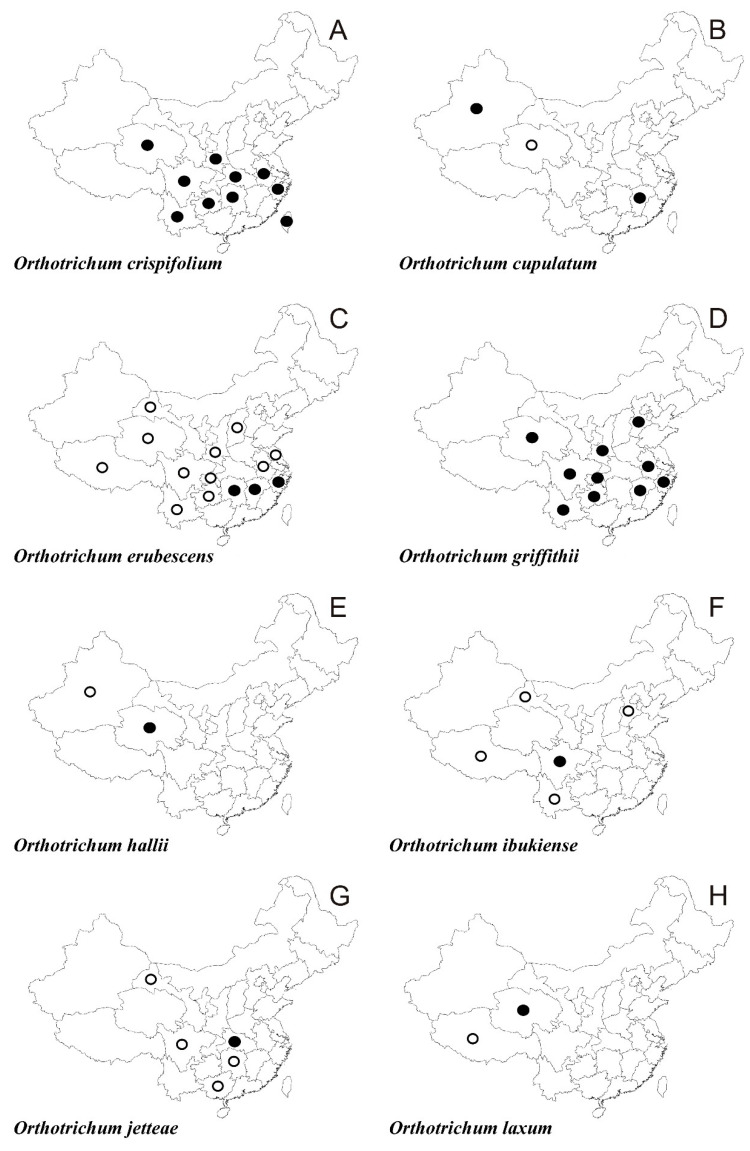
Distribution maps for species of the genus *Orthotrichum* in China’s provinces.

**Figure 6:**
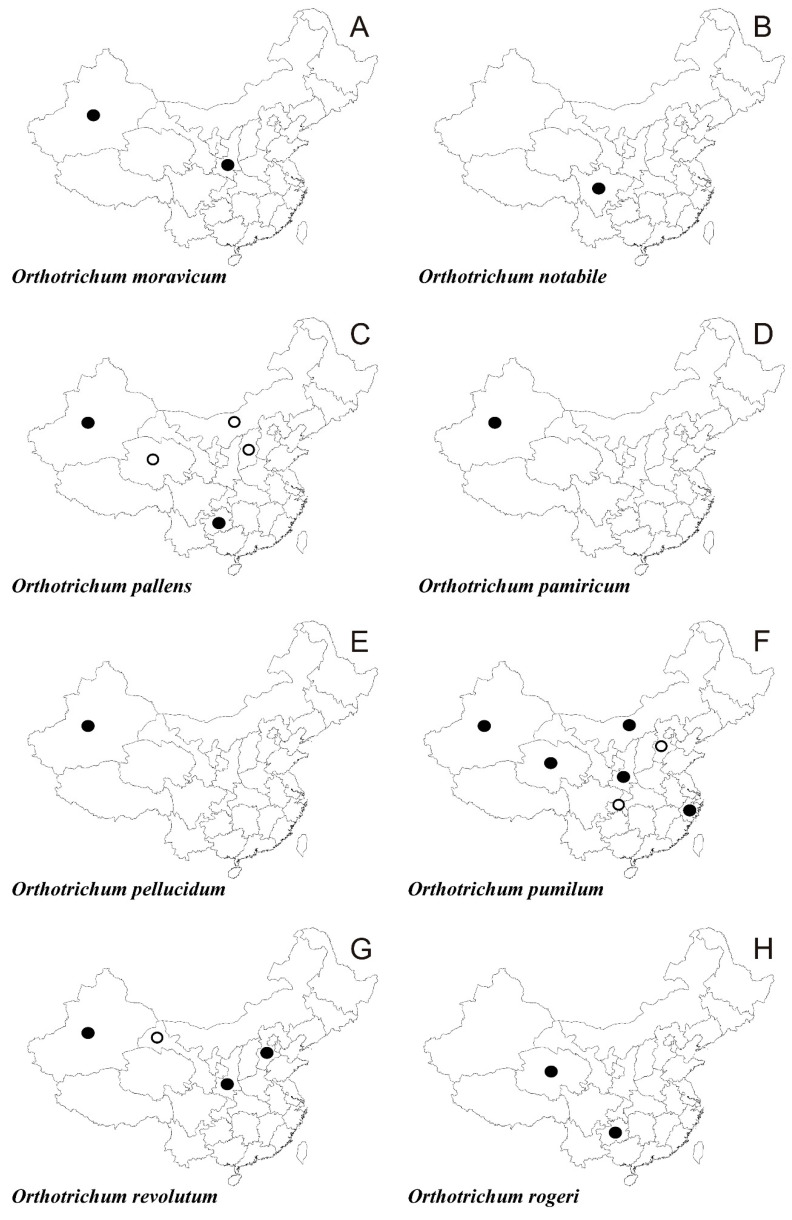
Distribution maps for species of the genus *Orthotrichum* in China’s provinces.

**Figure 7:**
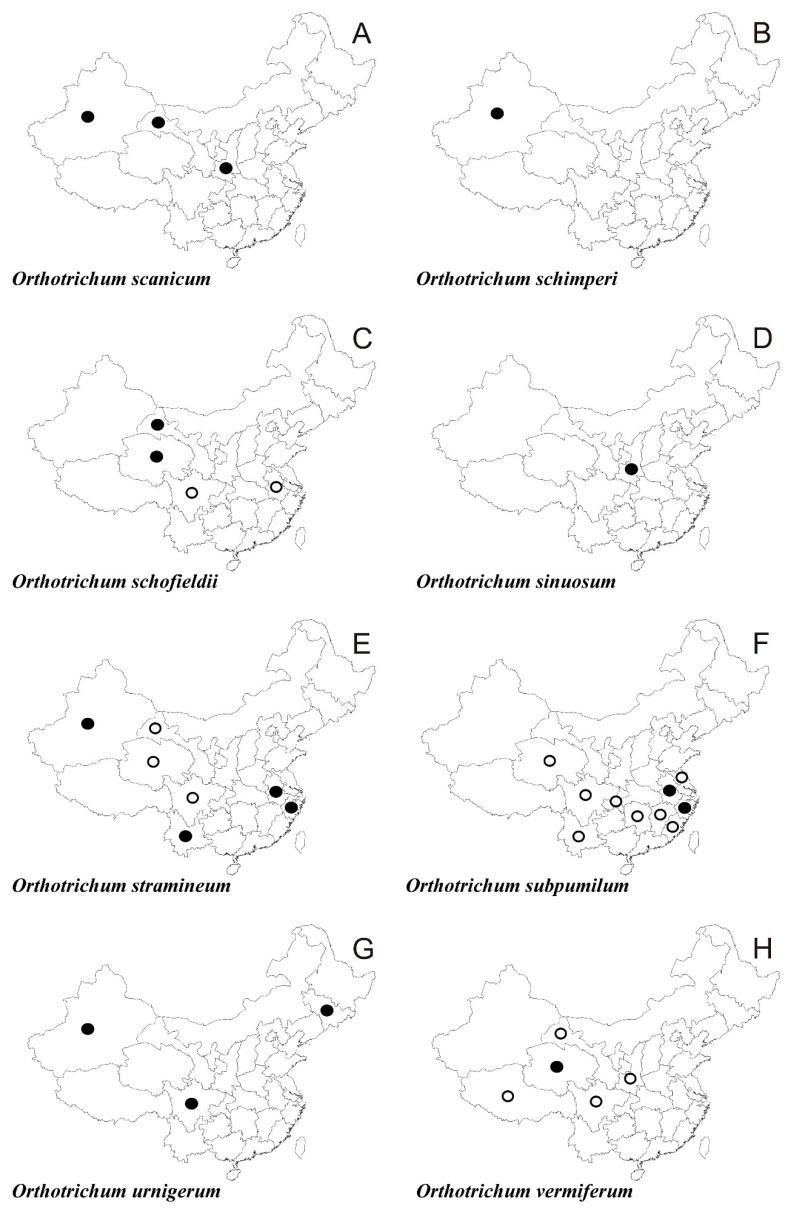
Distribution maps of species of the genus *Orthotrichum* in China’s provinces.

In the following synopsis all accepted taxa are arranged alphabetically by genus and then by species within genera. For each taxon all provinces in which the taxa concerned have been recorded are listed alphabetically and for each province all literature data, if available, are enumerated in chronological order. Provinces from which the herbarium material have been studied are listed separately. All specimens examined are presented in Supplementary Materials “S1”, including the personal collections of the first three authors. For some taxa species with taxonomic and/or distributional notes are added. The occurrence of all taxa of orthotrichalean mosses in provinces of China is presented in Table 2 and their distribution is mapped (Figure 2, Figure 3, Figure 4, Figure 5, Figure 6, Figure 7), including literature data (open circles) and specimen-based records (solid dots). Finally, a key to determination of all taxa of orthotrichalean mosses in China is provided.

### 3.1. Accepted Taxa

***Florschuetziella scaberrima*** (Broth.) Vitt (Figure 2A)

*Literature data:***Y [13,42,43,49,69]**.

*Note*: Despite its distinctiveness this species is one of the most puzzling and neglected species in the moss flora of China. It was originally described as a new species, *Orthotrichum scaberrimum*, from two collections made by Handel-Mazzetti from north-western Yunnan [40]. Vitt [43] briefly assessed this species taxonomically and concluded that the most appropriate home for it is in *Florschuetziella* Vitt, a monotypic genus consisting of only *F. steerei* Vitt known only from a single collection from the state of Chiapas in southern Mexico [100,101]. Accordingly, *O. scaberrimum* was transferred to this genus as *F. scaberrima*, although Vitt [43] stated that the Chinese and Mexican species are closely related and possibly they are conspecific. The genus *Florschuetziella* is currently placed in the tribe Macromitrieae in the subfamily Macromitrioideae of the family Orthotrichaceae [102]. The change of the taxonomic status of *O. scaberrimum* was overlooked by the compilers of the recent checklist of Chinese mosses [13] in which this species is still listed under its original name in *Orthotrichum*. Surprisingly, *O. scaberrimum* is not dealt with either in the moss Flora of Yunnan [103] or in the Chinese and English versions of the moss Flora of China [1,2]. As well, it has been omitted in subsequent catalogue of the bryophytes in China [3], nor was it mentioned in the newest survey of *Orthotrichum* in this country [14]. The species is still known only from the type material and any additional specimens have been collected during field studies and detected among holdings of orthotrichalean mosses in Chinese herbaria.

***Leratia exigua*** (Sull.) Goffinet (Figure 2B)

*Literature data:***C** [1,2], **F** [13,104,105], **Gh** [1,13,37,38,77,79,80,105], **Hu** [1,105], **Js** [1,2], **Jx** [1,2,12,13,105], **Sa** [94], **Si** [12,13,38,45,105], **Y** [1,2].

*Herbarium specimens examined*: **A**, **F**, **Gh**, **Hu**, **Jx**, **Sa**, **Si**, **Y**, **Z** (for details see Supplementary Materials “S1”).

***Lewinskya affinis*** (Brid.) F. Lara, Garilleti & Goffinet var. ***affinis*** (Figure 2C)

*Literature data:***C** [1,2,83], **Hl** [74], **I** [13,75,81,82,83,106], **Jl** [13,47,107,108], **L** [13,47,107], **N [82,83]**, **Sd** [64], **Si** [64,83], **Sx [13,109]**, **Xi** [1,2,13,59,83,110].

*Herbarium specimens examined:***A**, **Gh**, **I**, **Jl**, **Sd**, **Si**, **Xi**, **Z** (for details see Supplementary Materials “S1”).

***Lewinskya affinis*** var. ***bohemica*** (Plášek & Sawicki) Plášek (Figure 2D)

*Herbarium specimen examined*: **Xi** (for details see Supplementary Materials “S1”).

*Note*: New taxa for China.

**Figure 8:**
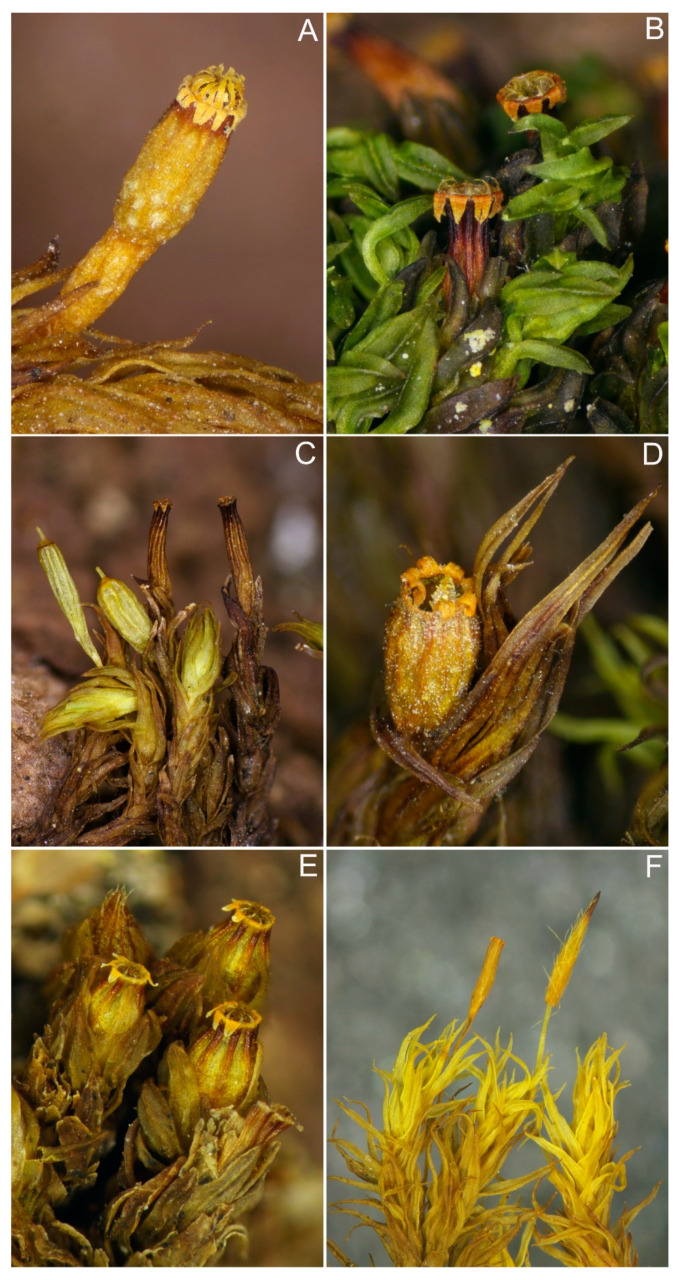
Macro photographs of some orthotrichalean species in China: (**A**) *Lewinskya dasymitria*, (**B**) *Orthotrichum griffithii*, (**C**) *Orthotrichum laxum*, (**D**) *Lewinskya erosa*, (**E**) *Orthotrichum erubescens*, (**F**) *Lewinskya hookeri* var. *hookeri*.

***Lewinskya dasymitria*** (Lewinsky) F. Lara, Garilleti & Goffinet (Figure 2E and Figure 8A)

*Literature data:***G** [1,14], **Gh** [77,79], **Hb** [14,64], **Jl** [14,64], **Q** [14,60,64], **Sa** [1,2], **Si** [1,2,12,13,14,52,55], **Sx** [12,13,14], **Xi** [1,64,111], **Xz** [1,2,3,12,13,14,52,112], **Y** [12,13].

*Herbarium specimens examined:***Gh**, **Hb**, **Jl**, **Q**, **Si**, **Sx**, **Xi**, **Xz**, **Y** (for details see Supplementary Materials “S1”).

***Lewinskya erosa*** (Lewinsky) F. Lara, Garilleti & Goffinet (Figure 2F and Figure 8D)

*Literature data:***G [14,96]**, **Gh [53]**, **Hb** [14,64], **Sa** [1,2,3,12,13,14], **Si** [14,54].

*Herbarium specimens examined:***G**, **Hb**, **Si** (for details see Supplementary Materials “S1”).

**Figure 9:**
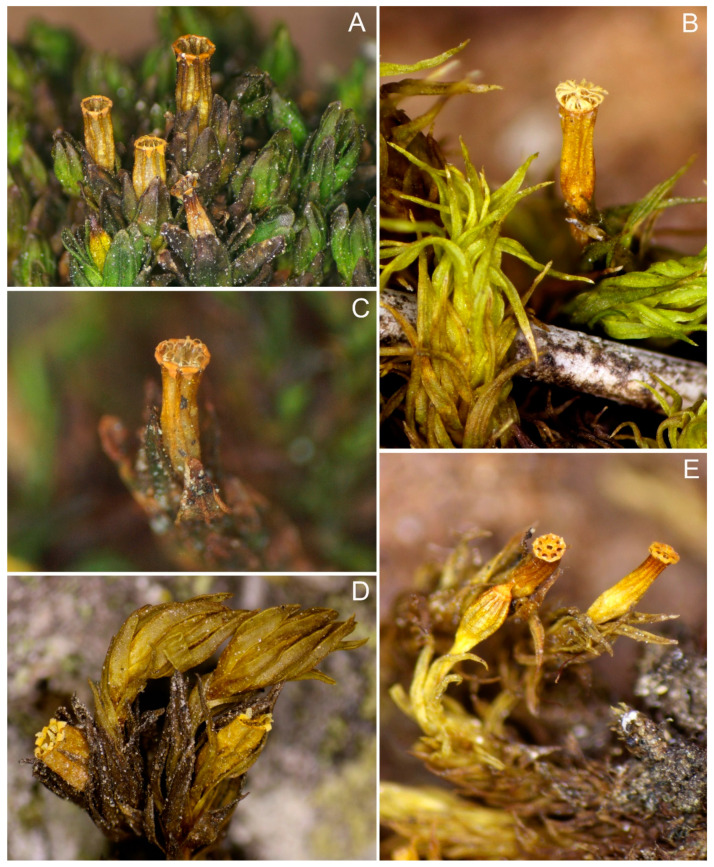
Macro photographs of some orthotrichalean species in China: (**A**) *Orthotrichum pamiricum*, (**B**) *Lewinskya pulchra*, (**C**) *Orthotrichum moravicum*, (**D**) *Lewinskya graphiomitria*, (**E**) *Orthotrichum callistomum*.

***Lewinskya graphiomitria*** (Müll. Hal. *ex* Beckett) F. Lara, Garilleti & Goffinet (Figure 2G and Figure 9D)

*Literature data:***Gh** [66], **Jx** [66], **Ta** [66].

*Herbarium specimens examined:***Gh**, **Jx**, **Ta** (for details see Supplementary Materials “S1”).

***Lewinskya hookeri*** var. ***granulata*** (Lewinsky) F. Lara, Garilleti & Goffinet (Figure 2H)

*Literature data:***G** [14], **Sa** [1,2,3,12,13,35,36,49], **Si** [1,2,12,13,14], **Xz** [1,2,12,13], **Y [1,2,12,13,14]**.

*Herbarium specimens examined:***Si**, **Y** (for details see Supplementary Materials “S1”).

***Lewinskya hookeri*** (Wilson *ex* Mitt.) F. Lara, Garilleti & Goffinet var. ***hookeri*** (Figure 3A and Figure 8F)

*Literature data:***C** [1,2], **G** [1,2,14,83], **Gh** [77,78,79], **Q [1,2,14,57]**, **Sa [14,83]**; **Si** [1,2,12,13,29,42,49,55,83], **Xi** [1,2,83], **Xz** [1,2,12,13,14,112], **Y** [1,2,12,13,14,31,42,49,67,103,112,113].

*Herbarium specimens examined:***G**, **Sa**, **Si**, **Xi**, **Xz**, **Y** (for details see Supplementary Materials “S1”).

***Lewinskya iwatsukii*** (Ignatov) F. Lara, Garilleti & Goffinet (Figure 3B)

*Literature data:***Gh** [77,78], **Q [64]**, **Si [1,2]**, **Xi [54,64]**, **Xz** [1,2,12,13,14,50], **Y** [1,2].

*Herbarium specimens examined:***Q**, **Xi**, **Y** (for details see Supplementary Materials “S1”).

*Note*: This species was recorded from China as *Orthotrichum laevigatum* var. *japonicum* [1,2] which is an Asian counterpart of the type variety being an Euro-North American taxon [114,115]. The Asian variety was subsequently raised to species as *O. iwatsukii* (≡*Lewinskya iwatsukii*) [51] and it is scattered in East Asia, including Japan, China, Nepal and NW India [12]. Although any records of the type variety of *O. laevigatum* have been published from Asia, in some Chinese herbaria there have been located many specimens so named from Xizang (XJU#14215; IFP#420; XJU#12811, 18465, 12913, 28066, 26500, 5953, 18075, 15304, 14073) and Yunnan (KUN#B0010584) which actually represent *Lewinskya iwatsukii*.

***Lewinskya leiolecythis*** (Müll. Hal.) F. Lara, Garilleti & Goffinet (Figure 3C)

*Literature data:***G [14]**, **Hb** [1,2,12,13], **I [14]**, **Jl [14]**, **Jx [14]**, **Q [14]**, **Sa** [1,2,3,12,13,14,33,36,49], **Si** [1,2,13,14], **Sx [14]**, **Xi [14]**, **Xz [14]**, **Y** [13,76,116].

*Herbarium specimens examined:***Sa** (for details see Supplementary Materials “S1”).

***Lewinskya pulchra*** (Lewinsky) F. Lara, Garilleti & Goffinet (Figure 3D and Figure 9B)

*Literature data:***Gh** [77,79], **Q [1,2]**, **Si** [1,2,3,12,13,14,55].

*Herbarium specimens examined:***Si** (for details see Supplementary Materials “S1”).

***Lewinskya rupestris*** (Schwägr.) F. Lara, Garilleti & Goffinet (Figure 3E)

*Literature data:***Hu** [105], **Sa [13,36]**, **Si [14]**, **Xi [1,2,12,13,14,53,59,83,110,117,118,119]**, **Xz** [14,105], **Y** [1,83].

*Herbarium specimens examined:***Si**, **Xi** (for details see Supplementary Materials “S1”).

*Note*: Redfearn et al. [13] recorded this species from Inner Mongolia after the work of Bai [120]; however, this species is not subsequently mentioned in the moss Flora of this province [81].

***Lewinskya sordida*** (Sull. & Lesq.) F. Lara, Garilleti & Goffinet (Figure 3F)

*Literature data:***C** [1,2,14,83], **Hb** [1,13,14,83], **Hl** [64], **I** [1,2,12,13,14,81,83,106], **Jl [1,2,12,13,14,83,121]**, **N [82,83]**, **Sa [14]**, **Si [14]**, **Xi** [1,13,53,59,83], **Y [14]**, **Z [86]**.

*Herbarium specimens examined:***Hb**, **Hl**, **I**, **Jl**, **Xi** (for details see Supplementary Materials “S1”).

***Lewinskya speciosa*** (Nees) F. Lara, Garilleti & Goffinet (Figure 3G)

*Literature data:***C** [1,2,83], **G** [64], **Hb** [64], **He** [98], **Hl** [13,14,83,107], **Hu [64]**, **I** [13,14,81,82,83,106,120,122], **Jl** [1,2,12,13,47,83,107,121], **L [13,47,83,107]**, **Q** [1,2,14,57,83], **Si** [1,83], **Sx** [13,123], **Xi** [1,12,13,14,53,59,83,110,119], **Xz** [14], **Y** [1,2,83], **Z** [13,86,124,125].

*Herbarium specimens examined:***C**, **G**, **Hb**, **He**, **Hl**, **Hu**, **I**, **Jl**, **Q**, **Sa**, **Si**, **Sx**, **Ta**, **Xi**, **Xz**, **Y** (for details see Supplementary Materials “S1”).

***Lewinskya striata*** (Hedw.) F. Lara, Garilleti & Goffinet (Figure 3H)

*Literature data:***G [14,99]**, **Hb** [64], **Hl [13,107]**, **I** [81,106], **Jl** [1,2,12,13,47,107,121], **Jx [14]**, **L** [13,47,107], **N [82]**, **Sa** [14,98], **Si** [1,2,13,14,46,52], **Sx [13,123]**, **Xi [13,53,59,110,119]**, **Xz** [1,2], **Y [13,14,113]**.

*Herbarium specimens examined:***G**, **Hb**, **Jl**, **Jx**, **Sa**, **Si**, **Sx**, **Xi**, **Xz** (for details see Supplementary Materials “S1”).

***Lewinskya taiwanensis*** (Lewinsky) F. Lara, Garilleti & Goffinet (Figure 4A)

*Literature data:***Gh [1,2,77,79,80,83]**, **Ta** [1,2,3,12,13,83], **Xz** [1,2,83].

*Herbarium specimens examined:***Ta** (for details see Supplementary Materials “S1”).

***Lewinskya vladikavkana*** (Venturi) F. Lara, Garilleti & Goffinet (Figure 4B)

*Literature data:***Sa** [64], **Si** [64], **Sx** [64], **Xi** [64], **Xz** [64], **Y** [64].

*Herbarium specimens examined:***Q**, **Sa**, **Si**, **Sx**, **Xi**, **Xz**, **Y** (for details see Supplementary Materials “S1”).

***Nyholmiella obtusifolia*** (Brid.) Holmen & E. Warncke (Figure 4C)

*Literature data:***G** [12,13,85], **He** [98], **Hl [1,2,12,13]**, **I [1,2,12,13,81,106,120]**, **Jl [13,47,107]**, **Jx [1,2]**, **L** [13], **N [82]**, **Q** [1,2,57], **Sd** [64], **Si [1,2]**, **Xi [1,2,13,53,59,110,119]**, **Y** [1,2,103].

*Herbarium specimens examined:***G**, **He**, **Hl**, **I**, **L**, **Q**, **Sa**, **Sd**, **Si**, **Xi**, **Y** (for details see Supplementary Materials “S1”).

***Orthotrichum alpestre*** Wilson (Figure 4D)

*Literature data:***G** [64], **Xi** [64], **Xz [14]**.

*Herbarium specimens examined:***G**, **Xi** (for details see Supplementary Materials “S1”).

***Orthotrichum anomalum*** Hedw. (Figure 4E)

*Literature data:***F [1,2]**, **Gh [77,79]**, **He [1,2,14]**, **Hl** [64], **I** [1,2,13,14,81,82,83,106,120], **N [82]**, **Q** [1,2,14,57,83], **Sa** [13,48,126], **Sd [14]**, **Si [1,2,14,83]**, **Sx** [13,14,83,109], **Xi** [1,2,13,14,53,59,83,110,119,127], **Xz** [1,2,12,13,14,50,83], **Y** [1,2,12,13,83,103].

*Herbarium specimens examined:***Gh**, **He**, **Hl**, **I**, **Jx**, **Q**, **Si**, **Sx**, **Xi**, **Xz**, **Y** (for details see Supplementary Materials “S1”).

***Orthotrichum callistomum*** Fisch.-Oost. *ex* Bruch & Schimp. (Figure 4F and Figure 9E)

*Literature data:***G** [14,96], **Gh** [77,78,79], **Q** [1,2,14,57], **Si [1,2,12,13,14,41,42,55,112,128]**, **Sx [14]**, **Ta** [12,13,72,128,129,130,131,132], **Xz** [12,13,14,128], **Y** [1,2,3,12,13,14,41,42,69,103,112,128].

*Herbarium specimens examined:***G**, **Sa**, **Si**, **Xz**, **Y** (for details see Supplementary Materials “S1”).

***Orthotrichum consobrinum*** Cardot (Figure 4G)

*Literature data:***A [1,2,12,13,40,133]**, **G [1,2,14]**, **Gh** [77,78,79], **Gx** [134,135,136], **Hb** [13,40,42,133], **Hu [1,2,12,13,14,40,133]**, **Js** [1,2,12,13,14,40,73,133], **Jx** [13,14,137], **Sh** [1,2,3,12,13,14,39,40,73,133,138], **Y [1,2,103,139]**, **Z** [13,14,40,86,124,133].

*Herbarium specimens examined:***A**, **Gh**, **Hu**, **Js**, **Sa**, **Sh**, **Y**, **Z** (for details see Supplementary Materials “S1”).

***Orthotrichum crenulatum*** Mitt. (Figure 4H)

*Literature data:***I** [14,64], **Xi** [2,14,59,83].

*Herbarium specimens examined:***I**, **Xi** (for details see Supplementary Materials “S1”).

*Note*: As indicated in the earlier section of this paper, *Orthotrichum crenulatum* was described from material collected in the Shyok River Valley, a tributary of the Indus River that flows through northern Ladakh in India and enters the region of Gilgit-Baltistan in Pakistan where Th. Thomson collected this moss in the Rondu District. Hence, the records of this species from Xizang cited by Lewinsky [12], Redfearn et al. [13], and Jia et al. [2] are incorrect and should be deleted, as is the case with the citation of the type of *O. crenulatum* to be collected in China [14]. Actually, this species was collected for the first time in China from Xinjiang Province [59].

***Orthotrichum crispifolium*** Broth. (Figure 5A)

*Literature data:***Hb** [64], **Hu** [64], **Si** [64], **Y [1,2,3,12,13,42,69,103]**, **Z [64]**.

*Herbarium specimens examined:***A**, **Gh**, **Hb**, **Hu**, **Q**, **Sa**, **Si**, **Ta**, **Y**, **Z** (for details see Supplementary Materials “S1”).

***Orthotrichum cupulatum*** Brid. (Figure 5B)

*Literature data:***Q** [14,57], **Xi** [13,14,53,59].

*Herbarium specimens examined:***Jx**, **Xi** (for details see Supplementary Materials “S1”).

***Orthotrichum erubescens*** Müll. Hal. (Figure 5C and Figure 8E)

*Literature data:***A** [1,2,12,13,14], **C [1,2,14]**, **G [14]**, **Gh** [1,3,12,13,37,76,77,79,80], **Hu** [1,2,12,13,14], **Js** [12,13], **Jx [14,64]**, **Q** [61], **Sa** [1,3,12,13,34,36], **Sx [14]**, **Si [14]**, **Xz [14]**, **Y [14]**, **Z [14]**.

*Herbarium specimens examined*: **Hu**, **Jx**, **Z** (for details see Supplementary Materials “S1”).

**Orthotrichum griffithii** Mitt. ex Dixon (Figure 5D and Figure 8B)

*Literature data:***Si [1,2]**, **Y** [1,2,12,13,103,113].

*Herbarium specimens examined:***A**, **C**, **Gh**, **He**, **Jx**, **Q**, **Sa**, **Si**, **Y**, **Z** (for details see Supplementary Materials “S1”).

***Orthotrichum hallii*** Sull. & Lesq. (Figure 5E)

*Literature data:***Xi** [1,2,13,53,54,59,111].

*Herbarium specimen examined*: **Q** (for details see Supplementary Materials “S1”).

***Orthotrichum ibukiense*** Toyama (Figure 5F)

*Literature data:***G** [1,2,83], **He** [1,2,83], **I [2]**, **Si** [1,2,83], **Xi [2]**, **Xz** [1,2], **Y** [140].

*Herbarium specimens examined:***Si** (for details see Supplementary Materials “S1”).

***Orthotrichum jetteae*** B. H. Allen (Figure 5G)

*Literature data:***G [14]**, **Gh** [1,3,77,79,89,141,142], **Hu** [1,79,105,141,142], **Si [14]**.

*Herbarium specimen examined*: **Hb** (for details see Supplementary Materials “S1”).

***Orthotrichum laxum*** Lewinsky (Figure 5H and Figure 8C)

*Literature data:***Q** [1,3,60], **Xz [14]**.

*Herbarium specimen examined*: **Q** (for details see Supplementary Materials “S1”).

***Orthotrichum moravicum*** Plášek & Sawicki (Figure 6A and Figure 9C)

Literature data: **Xi [65]**.

*Herbarium specimens examined*: **Sa**, **Xi** (for details see Supplementary Materials “S1”).

***Orthotrichum notabile*** Lewinsky (Figure 6B)

*Literature data:***Si** [1,3,55,56].

*Herbarium specimens examined:***Si** (for details see Supplementary Materials “S1”).

*Note*: Wang and Jia [14] considered *Orthotrichum notabile* to be conspecific with *O. stramineum* because they stated that the two species shared some characters including erect-appressed leaves with ovate bases, smooth, linear processes, often semi-immersed stomata and glabrous calyptrae. They considered *O. notabile* to be an aberrant form of *O. stramineum* with larger spores (30–36 μm versus 15–22 μm in diameter) owing to being in the phase of germination. However, in the opinion of the authors of this article, both species are clearly defined and significantly different. *Orthotrichum notabile* can be distinguished from *O. stramineum* mainly by the presence of eight segments of the endostome (*O. stramineum* has 16) and naked vaginula (in *O. stramineum* the vaginula is always covered by noticeably long hairs).

***Orthotrichum pallens*** Brid. (Figure 6C)

*Literature data:***Q** [57], **I [14]**, **Sx [14]**, **Xi** [13,14,53,59].

*Herbarium specimens examined:***Gh**, **Xi** (for details see Supplementary Materials “S1”).

***Orthotrichum pamiricum*** Plášek & Sawicki (Figure 6D, 9A)

Literature data: **Xi** [63].

*Herbarium specimens examined:***Xi** (for details see Supplementary Materials “S1”).

***Orthotrichum pellucidum*** Lindb. (Figure 6E)

Literature data: **Xi** [54].

*Herbarium specimens examined:***Xi** (for details see Supplementary Materials “S1”).

***Orthotrichum pumilum*** Sw. (Figure 6F)

*Literature data:***C** [1,83], **He [1,2,12,13,14,83]**, **I** [14,64], **Q** [1,2,14,57,83], **Sa** [12], **Xi** [14,64,83].

*Herbarium specimens examined*: **I**, **Q**, **Sa**, **Xi**, **Z** (for details see Supplementary Materials “S1”).

***Orthotrichum revolutum*** Müll. Hal. (Figure 6G)

*Literature data:***G** [1,2], **Sa** [1,2,3,12,13,34,36].

*Herbarium specimens examined:***He**, **Sa**, **Xi** (for details see Supplementary Materials “S1”).

***Orthotrichum rogeri*** Brid. (Figure 6H)

Literature data: **Q** [65].

*Herbarium specimens examined:***Gh**, **Q** (for details see Supplementary Materials “S1”).

***Orthotrichum scanicum*** Grönvall (Figure 7A)

Literature data: **Xi [14,65]**.

*Herbarium specimens examined:***G**, **Sa**, **Xi** (for details see Supplementary Materials “S1”).

***Orthotrichum schimperi*** Hammar (Figure 7B)

*Herbarium specimens examined:***Xi** (for details see Supplementary Materials “S1”).

*Note*: New taxa for China.

***Orthotrichum schofieldii*** (B. C. Tan & Y. Jia) B. H. Allen (Figure 7C)

*Literature data:***A [14]**, **G** [14,95], **Q [1,3,14,57,61]**, **Si [14]**.

*Herbarium specimens examined:***G**, **Q** (for details see Supplementary Materials “S1”).

***Orthotrichum sinuosum*** Lewinsky (Figure 7D)

*Literature data*: **Sa** [3,12,13].

*Herbarium specimens examined:***Sa** (for details see Supplementary Materials “S1”).

***Orthotrichum stramineum*** Brid. (Figure 7E)

*Literature data:***G [14]**, **Q** [14,61], **Si [14]**, **Xi** [97], **Y [14,56,60]**.

*Herbarium specimens examined:***A**, **Xi**, **Y**, **Z** (for details see Supplementary Materials “S1”).

***Orthotrichum subpumilum*** E. B. Bartram *ex* Lewinsky (Figure 7F)

*Literature data:***A** [1,2,13,14], **C [14]**, **F [14], Hu** [14,105], **Js [2,3]**, **Jx** [1,12,13,14], **Q** [1,2,57], **Si [14]**, **Y [14]**.

*Herbarium specimens examined:***A**, **Z** (for details see Supplementary Materials “S1”).

***Orthotrichum urnigerum*** Myrin (Figure 7G)

*Literature data:***Jl** [64], **Si** [93], **Xi** [13,44,53,93,110,119].

*Herbarium specimens examined:***Jl**, **Si**, **Xi** (for details see Supplementary Materials “S1”).

***Orthotrichum vermiferum*** Lewinsky (Figure 7H)

*Literature data:***G [14]**, **Q** [1,3,14,61], **Sa [14]**, **Si [14]**, **Xz** [14].

*Herbarium specimens examined:***Q** (for details see Supplementary Materials “S1”).

### 3.2. Doubtful, Uncertain and Excluded Taxa

***Lewinskya brassii*** (E. B. Bartram) F. Lara, Garilleti & Goffinet

Literature data: **Xz** [1,2].

Note: The voucher herbarium specimen of this record (*M.-Z. Wang 12518b*, PE) is sterile and it rather represents a species of the genus *Zygodon*, not *Orthotrichum* Accordingly, this New Guinean species is excluded from a list of Chinese bryophytes.

***Lewinskya elegans*** (Hook. & Grev.) F. Lara, Garilleti & Goffinet

Literature data: **Xi** [53].

*Note*: During the study of extensive material of the genus *Lewinskya* from Asia (Tajikistan, Kyrgyzstan, Kazakhstan, China), we have not recorded a single specimen that would correspond to the species *Lewinskya elegans*. Similarly, Lara et al. [10] stated that this species is widespread only on the continent of North America. *Lewinskya elegans* most likely does not occur in China and until the specimens which could match well North American plants are found, the species is excluded from a list of China’s bryophytes.

***Nyholmiella gymnostoma*** (Brid.) Holmen & E.Warncke

Literature data: **E** [62].

*Note*: This species has only once been recorded by Gao [62] as “growing on branches or at the fork of branches in Eastern Provinces of China” but without citation of any specimens and/or giving specific localities. It was briefly described and illustrated with line drawing showing two leaves, gemma, mid-leaf cells at margins and stoma. Additionally, a colorful photograph showing the plants growing on a twig is added. However, the presented illustrations do not correspond to *N. gymnostoma* and differ mainly in the shape of the leaves that are without the hood-shaped apex and by presence of only one central papilla on each leaf cell.

***Orthotrichum hooglandii*** E. B. Bartram

Literature data: **Xi** [1,2].

Note: A revision of the herbarium specimen collected from Xinjiang by *J.-C. Zhao 1141* (HBNU) and reported by Jia et al. [1,2] as *Orthotrichum hooglandii* was not be possible because it has not been located and available for examination. In addition, the appearance of this tropical species which is known from New Guinea only in continental Asia is quite unlikely, so its occurrence in China is considered to be doubtful until the convincing voucher material is studied.

### 3.3. Key to Genera, Species and Varieties of Orthotrichalean Mosses in China

**1a.** Stomata cryptoporous (***Orthotrichum***)5**1b.** Stomata phaneroporous2**2b.** Dioicous; gemmae always present
***Nyholmiella obtusifolia***
**2a.** Monoicous; gemmae mostly absent3**3a.** Plants creeping, producing many erect-ascending branches
***Florschuetziella scaberrima***
**3b.** Plants erect-ascending, simple or forked4**4a.** Leaves oblong-lanceolate to oblong, decurrent
***Leratia exigua***
**4b.** Leaves acuminate, nondecurrent (***Lewinskya***)33

**5–32 *Orthotrichum***
**5a.** Capsules constricted and strongly plicate at mouth, exostome teeth rudimentary or absent6**5b.** Capsules non-plicate at mouth, exostome teeth well-developed7**6a.** Endostome segments 8, filiform, spores small, unicellular, 14-30 µm wide
***O. schofieldii***
**6b.** Endostome segments 16, broad and truncated, spores large multicellular,35-60 µm wide
***O. jetteae***
**7a.** Exostome teeth erect to spreading when dry, mostly epilithic species8**7b.** Exostome teeth reflexed or recurved when dry, mostly epiphytic species13**8a.** Laminal cells bistratose, at least in the upper part9**8b.** Laminal cells always unistratose10**9a.** Leaf lamina almost completely bistratose, papillae low and simple
***O. hallii***
**9b.** Leaf lamina rather unistratose with numerous bistratose patches, papillae high, clavate or forked
***O. pellucidum***
**10a.** Capsules exserted on long setae
***O. anomalum***
**10b.** Capsules immersed to emergent, setae short11**11a.** Capsules ovoid to urceolate, 16-ribbed when dry12**11b.** Capsules cylindric, 8-ribbed when dry
***O. ibukiense***
**12a.** Vaginula densely hairy with long hairs, exostome teeth roughly papillose
***O. urnigerum***
**12b.** Vaginula naked, exostome teeth finely papillose-striated
***O. cupulatum***
**13a.** Endostome segments broad, as wide as exostome teeth, united in upper parts and forming a perforated dome
***O. callistomum***
**13b.** Endostome segments narrower than exostome teeth, free14**14a.** Leaf margins plane or only slightly recurved near the base15**14b.** Leaf margins recurved or revolute almost throughout21**15a.** Leaves very often keeled16**15b.** Leaves not keeled17**16a.** Leave strongly crisped when dry, usually keeled in the central part
***O. crispifolium***
**16b.** Leave undulate and flexuose when dry, usually keeled at the base
***O. griffithii***
**17a.** Leaves at least in the apices conspicuous undulate or flexuose when dry18**17b.** Leaves straight or only slightly undulate or flexuose when dry19**18a.** Leaves undulate and flexuose when dry, with rounded-acute apices, endostome segments half of the height of the teeth
***O. sinuosum***
**18b.** Leaves flexuose only at the apices when dry, with acute apices, endostome segments almost as tall as teeth
***O. consobrinum***
**19a.** Leaves fromovate, somewhat concave bases rather suddenly tapering to the apices
***O. notabile***
**19b.** Leaves gradually tapering to the apices20**20a.** Leaves narrowly lanceolate, endostome segments with a smooth outer surface, not united at the base, perichaetial leaves acute
***O. subpumilum***
**20b.** Leaves lanceolate, endostome segments remarkably ornamented with vertical striate on the outer surface, sometimes united by broader bases, perichaetial leaves obtuse
***O. erubescens***
**21a.** Endostome segments 1622**21b.** Endostome segments 829**22a.** Vaginula hairy23**22b.** Vaginula naked24**23a.** Endostome segments alternately longer and shorter (the intermediate ones shorter than the primary), calyptra hairy
***O. stramineum***
**23b.** Endostome segments of the same length, calyptra naked
***O. vermiferum***
**24a.** Endostome segments with lateral appendages25**24b.** Endostome segments without lateral appendages27**25a.** Appendages conspicuously long (the segments resembls a branched tree), often with appendiculae linking contiguous segments
***O. moravicum***
**25b.** Appendages short26**26a.** Exostome teeth 8, endostome segments 16, calyptra naked, margins at leaf apex entire
***O. revolutum***
**26b.** Exostome teeth 16, endostome segments 16, calyptra sparsely hairy, margins at leaf apex often with a few irregular teeth
***O. scanicum***
**27a.** Leaves ovate to ovate-lanceolate, with obtuse apex
***O. pamiricum***
**27b.** Leaves oblong-lanceolate to lanceolate, acute to rounded-acute at the apex28**28a.** Endostome formed by 16 well developed narrow segments, calyptra naked
***O. pallens***
**28b.** Endostome formed by 8 well developed stout segments and 8 rudimentary ones, calyptra hairy
***O. laxum***
**29a.** Vaginula hairy, leaf cells with prominent and commonly forked papillae
***O. alpestre***
**29b.** Vaginula naked, leaf cells smooth or with low simple papillae30**30a.** Endostome segments completely erect when dry, slightly striate at base; plants showing dimorphism of branch leaves including ligulate ones with a broadly oval base and margin recurved almost throughout on branches producing archegonia and significantly smaller ones, with margins plane or just a little recurved in the middle part on branches producing antheridia
***O. rogeri***
**30b.** Endostome segments incurved when dry, smooth or papillose; no leaf dimorphism present31**31a.** Leaves ligulate to ovate, obtuse to rounded-acute, sometimes partially bistratose
***O. crenulatum***
**31b.** Leaves ovate-lanceolate, acute to rounded-acute, always unistratose32**32a.** Capsule ovoid or pyriform when moist, abruptly contracted into seta, segments conspicuous broadly at base, 1/2 to 3/4 as long as the exostome teeth
***O. schimperi***
**32b.** Capsule oblong-cylindrical when moist, gradually contracted into seta, segments linear, almost as long as the exostome teeth
***O. pumilum***



**33–45 *Lewinskya***
**33a.** Exostome teeth erect to spreading when dry34**33b.** Exostome teeth reflexed, recurved or revolute when dry35**34a.** Capsules immersed, furrowed when dry; endostome rudimental or lacking; leaves partially or completely bistratose
***L. rupestris***
**34b.** Capsules long-exserted, smooth when dry, endostome well developed, leaves unistratose
***L. iwatsukii***
**35a.** Capsules short to long-exserted36**35b.** Capsules immersed of emergent40**36a.** Capsules long–exserted, setae 4-15 mm long37**36b.** Capsules short–exserted, setae to 2 mm long38**37a.** Leaves strongly contorted and twisted when dry, setae ca. 4 mm long
***L. taiwanensis***
**37b.** Leaves erect or flexuose when dry, setae up to 15 mm long
***L. hookeri***
**37ba.** Spores roughly papillose, usually 37-53 µm wide***L. hookeri*** var. ***hookeri*****37bb.** Spores finely papillose, usually 25-40 µm wide***L. hookeri*** var. ***granulatum*****38a.** Peristome consisting of 16 exostome teeth and 16 endostome segments, leaf margin plane
***L. pulchra***
**38b.** Peristome consisting of 8 pairs of exostome teeth and 8 endostome segments, leaf margin recurved39**39a.** Endostome segments broad, each as wide as a tooth of each exostome pair
***L. vladikavkana***
**39b.** Endostome segments slender, linear, exostome teeth perforate to the base and cancellate near the apex
***L. sordida***
**40a.** Capsules smooth when dry41**40b.** Capsules furrowed at least in the upper half when dry43**41a.** Endostome segments 16L. striata**41b.** Endostome segments 8 42**42a.** Endostome broadly triangular with conspicuous appendages in the margins
***L. erosa***
**42b.** Endostome narrowly triangular without the appendages
***L. graphiomitria***
**43a.** Capsules only slightly furrowed when dry44**43b.** Capsules strongly furrowed when dry
***L. affinis***
**42ba.** Endostome segments 8***L. affinis*** var. ***affinis*****42bb.** Endostome segments 16***L. affinis*** var. ***bohemica*****44a.** Endostome segments 845**44b.** Endostome segments 16, capsules with up to conspicuous 8 or more red to reddish brown rings of thick-walled cells below the mouth
***L. dasymitria***
**45a.** Capsules long cylindric, with 8 noticeable furrows in distal half part, calyptra densely hairy with long hairs, endostome segments coarsely papillose
***L. speciosa***
**45b.** Capsules obloid to ovoid, only slightly furrowed below the mouth, calyptra sparsely hairy with short hairs or naked, endostome segments only finely papillose
***L. leiolecythis***



## 4. Discussion

### 4.1. Diversity of Orthotrichalean Mosses in China

Due to their unique morphology, especially that of sporophytes, orthotrichalean mosses are very clearly distinguishable from all other mosses and have always been unmistakably classified into the family of their own, Orthotrichaceae, one of the earliest recognised moss families [143]. In the course of about 130 years of bryological exploration, in the years 1892–2020, 59 species and two varieties as well as two *nomina nuda* have been reported in the literature from China, together with *Lewinskya affinis* var. *bohemica* and *Orthotrichum schimperi* which are recorded here for the first time from China (Table 1). The vast majority of these taxa were reported under the generic name *Orthotrichum*, with one species given a name under *Racomitrium* Brid. Two species have recently been placed in the new genus *Orthomitrium* and two species and one variety have been recorded as members of the genus *Lewinskya*. In modern moss taxonomy they represent 46 species and two varieties belonging to five genera including *Florschuetziella*, *Leratia*, *Nyholmiella* each consisting of one species in China, *Lewinskya* with 14 species and two varieties, and *Orthotrichum* comprising 29 species. *Florschuetziella* is classified in the tribe Macromitrieae of the subfamily Macromitrioideae, whereas the remaining genera belong to the subfamily Orthotrichoideae. Of these, *Leratia* is a member of the tribe Zygodonteae and *Nyholmiella*, *Lewinskya* and *Orthotrichum* are members of the tribe Orthotricheae [102]. The members of these four genera constitute the traditionally broadly circumscribed genus *Orthotrichum* as adopted in Chinese Floras and surveys of the Orthotrichaceae [1,2,14].

Considering all accepted taxa in the present treatment, the broadly conceived genus *Orthotrichum* consists currently of 46 species and two varieties in China. This represents a remarkable increase of the diversity of the orthotrichalean mosses in China, which is partly a result of many bryological exploratory expeditions to various parts of the country during recent years and, in part, of a critical revision of the herbarium holdings of orthotrichalean mosses deposited in various Chinese herbaria.

The diversity of *Orthotrichum s. lato* in China is the greatest among all Asian countries. A similar species richness of this genus is only known from Japan, but there only 37 species have been detected so far [144]. In the Asian part of Russia, almost half the number of species have been found (24 spp.), including nine species in *Lewinskya*, 14 in *Orthotrichum* and one in *Nyholmiella* [145]. The same number of species has so far been recorded from Kazakhstan [97,98], whereas 22 species have been discovered in Kyrgystan [97] and 18 in Tajikistan [146]. Other neighboring countries of China have far less species diversity of this genus, although in part this may be the result of understudy of the bryoflora. Only five species are known from Korea [147], 13 from India [148], ten from Mongolia [149], ten species and two varieties from Afghanistan [150], eight from Pakistan [12,151], four species and one variety from Bhutan [12], and two species and one variety from Nepal [12]. In Myanmar no species of this genus have so far been detected [152] and it is likely that here and in neighbouring countries, lack of comprehensive surveys may account for the lack of species.

In the continental context, the diversity of *Orthotrichum s. lato* in China is only smaller by 12 species from that in Europe where 57 species are known to occur [153] and by six species from that in North America including Mexico where 51 species have hitherto been discovered [101,114], but it is much larger than in remaining continents, although judging from some recent discoveries the diversity of the genus has still not been fully documented. Thus, in South America some 35 species have been recorded [154,155,156], in Central America six species [73], in sub-Saharan Africa ten species [14,157], in Australasia [158] nine species, and in the Antarctic only one species [159].

The vast territory of China is extremely diversified geologically, topographically and above all climatically both in the meridional and latitudinal gradients which gave rise to the division of the territory of China into seven phytogeographical areas [160]. It is perfectly reflected in the geographical distribution and floristic richness of individual provinces and this is clearly visible in the case of orthrotrichalean mosses (Table 2). The greatest species richness is found in eight provinces in the western and central part of the country. Hitherto, 27 species have been found in Sichuan and Xinjiang, 24 in Qinghai and Shaanxi, 21 in Yunnan, 19 in Gansu, and 18 in Guizhou and Xizang. In turn, 11 provinces in eastern China have average species richness ranging from six to 11 species. So far, 11 species have been found in Inner Mongolia and Zhejiang, ten in Jiangxi and Shanxi, nine in Anhui, Hubei and Chonhqing, eight in Hunan and Jilin, seven in Hebei and only six in Heilongjiang. However, no taxa of orthotrichalean mosses have been found so far in three provinces (Guangdong, Henan and Hainan). Finally, the remaining eight provinces show very little species richness in this group, ranging from one (Guangxi and Shanghai) to five species (Ningxia and Taiwan). The above numbers do not seem to be final and future field studies will probably result in further discoveries of species from this taxonomically difficult group.

### 4.2. Phytogeographical Elements

Like other groups of plants, fungi and algae, the taxa of bryophytes can be grouped into phytogeographical elements whose accurate and precise definition is of paramount importance for an adequate designation of the regional affinities of the flora of a given area and its comparison with the floras of different geographical regions. Various phytogeographical elements have been recognised for Chinese and Southeast Asian bryophytes [161,162,163,164,165,166,167,168,169,170,171,172]. However, so far no uniform system of phytogeographical elements has been developed for the entire Holarctic, in which almost the whole territory of China is situated, with the exception of its southernmost fringes, which belong to the Palaeotropics or constitute a transitional zone to this plant kingdom. In this study, the scheme proposed by Koponen and Piippo [170] was adopted in general outline, but with some slight modifications. Some elements, such as the panholarctic one are broadly interpreted without subdivisions on boreal, temperate and meridional as well as continuous or disjunct sub-elements since in some cases classifying species to a particular distribution patterns is not easy due to the lack of adequate complete distributional data. In general, Chinese orthotrichalean mosses are classified into eight phytogeographical elements, of which the Asian element is subdivided into three sub-elements (Table 3).

**China’s Endemic element**

*Florschuetziella scaberrima*
*Lewinskya dasymitria*
*L. erosa*
*L. leiolecythis*
*L. pulchra*
*L. taiwanensis*
*Orthotrichum jetteae*
*O. laxum*
*O. notabile*
*O. schofieldii*
*O. subpumilum*
*O. sinuosum*
*O. vermiferum*

Endemicity, i.e., the occurrence of taxa only in a single restricted geographical area, is a very important indicator of the distinctiveness of the flora of a given territory and is the most important and indispensable premise for any consideration of the origin and age of its flora. Therefore, it has an historical background and the age of the flora is the most important factor determining the richness of endemics, especially at the species level. China is a country with a very high level of endemism. The flora of this country comprises over 30,000 seed plant species and endemics account for over a half of this total number, with 15,103 species (52.1%) [83,170,171]. The moss flora of China is one of the richest in the world and consists of about 1950 species [3]. However, the real number of species is certainly much greater, as evidenced by the numerous newly described endemic species and genera of these plants, for example *Yunnanobryum rhyacophilum* Shevock, Ochyra, S.He & D.G.Long [172,173,174], *Schistidium riparium* H.H. Blom, Shevock, D.G. Long & Ochyra and *S. mucronatum* H.H. Blom, Shevock, D.G. Long & Ochyra [175], *Grimmia ulaandamana* J. Muñoz, C. Feng, X.L. Bai & J. Kou [176], *Bryoerythrophyllum pseudomarginatum* J. Kou, X.M. Shao & C. Feng [177], *Mawenzhangia* Enroth, Shevock and Ignatov [178], *Didymodon obtusus* J. Kou, X.M. Shao & C. Feng [179], *Rheoshevockia* Ignatov, W.Z.Ma & D.G.Long [180], and *Encalypta papillosa* C. Feng, J. Kou & B. Niu [181]. All these records of new species and genera indicate that, despite a long history of bryological exploration, China still holds remarkable undiscovered biodiversity, including many unique taxa of considerable evolutionary and biogeographical importance.

Unfortunately, the problem of endemic moss taxa in China has not yet been thoroughly summarised. One of the reasons hindering such development is the difference in the number of accepted species in the English version of *Moss Flora of China* and the latest catalogue of mosses of this country [3]. In the case of the family Orthotrichaceae, this difference amounts to 19 species, mainly due to the fact that the flora of China’s mosses does not include all the species described and/or reported from that country, and their taxonomic status requires a critical revision. Moreover, two genera, *Drummondia* Hook. and *Rhachithecium* Le Jolis, are not related to the Orthotrichaceae, but belong to the families of their own [102]. In these two treatments *Orthotrichum scaberrimum* is not recorded and yet it represents a distinct species and genus, *Florschuetziella scaberrima*, endemic to China. Such differences result in different percentages of endemic species. Thus, in *Moss Flora of China,* [2] there are 70 species from the Orthotrichaceae, including 23 endemics which constitutes 32.9% of their total number, while Jia and He [3] listed 87 species in the catalogue, including 20 endemics (23%).

All endemic species of Orthotrichacae represent three genera, two of which are segregates of the traditionally interpreted genus *Orthotrichum*, namely *Lewinskya* (5 spp.) and *Orthotrichum* (7 spp.), and the only species of the third genus *Florschuetziella* was also originally given a name in *Orthotrichum*. *Orthotrichum s. lato* appears to be one of the richest in endemic species of all moss genera in China. The genus *Macromitrium* Brid. as presented by Jia and He [3] also contains 13 endemics, but no fewer than eight of them have not been critically assessed since their inceptions, so their taxonomic status is still uncertain. Endemic species of the orthotrichalean mosses do not exhibit a clear distribution pattern in China and most appear to be randomly scattered throughout the country from the southern boreal zone through the temperate to the meridional (warm temperate–subtropical) zone. It should be added that *Lewinskya dasymitria* is included in this group, although actually it is a subendemic of China and has recently been found in the Altai in Russia. Yet, it has its main centre of occurrence in China and, additionally, the Russian and Chinese localities are situated in the same phytochorion in the Altai mountains which extend to parts of Russia, Kazakhstan, China (Xinjiang Province), and Mongolia.

**Panholarctic element**

*Lewinskya affinis*
*L. affinis var. bohemica*
*L. sordida*
*L. speciosa*
*L. striata*
*Nyholmiella obtusifolia*
*Orthotrichum alpestre*
*O. anomalum*
*O. pallens*
*O. pellucidum*
*O. pumilum*
*O. scanicum*
*O. schimperi*
*O. stramineum*

This element comprises 13 species and one variety that have continuous or strongly dissected and highly disjunct geographical ranges, yet they are known to occur in each continent of the Holarctic. Only three species, *Nyholmiella obtusifolia*, *Orthotrichum anomalum* and *O. speciosum* are widespread and locally frequent and abundant throughout all or most arctic, boreal and temperate regions of this biome. The global ranges of the remaining taxa are variously and usually strongly dissected and disjunct and sometimes limited to narrow areas in a given continent. For example, *O. scanicum* has its main centre of occurrence in Europe, ranging from the southern boreal zone in Scandinavia to the meridional zone in North Africa, whereas in north America it is known only from a single record in the Atlantic part of the continent and in Asia it is widely scattered in Arctic Siberia and Central Asia [65]. In contrast, *Lewinskya sordida* is an arctic-boreal species in North America [182], in Europe it is known only from Spitsbergen [183] and the Caucasus and in Asia it is widespread in the Russian Far East [145], extending through Japan and Korea to Yunnan in China (Figure 3F) [12].

**Eurasian element**

*Lewinskya iwatsukii*
*L. vladikavkana*
*Orthotrichum callistomum*
*O. crenulatum*
*O. moravicum*
*O. rogeri*
*O. urnigerum*

The Eurasian element comprises a small group of seven disjunct species found mainly in Europe and appearing at highly disjunct stations in Central and East Asia. Two of these species, *Orthotrichum rogeri* and *O. urnigerum*, are fairly widely distributed in Western and Central Europe, southern Scandinavia, the Mediterranean and the Caucasus, and are very rare in Asia in a few widely scattered sites. In contrast, *O. callistomum* and *O. crenulatum* are exceedingly rare in the Alps and Caucasus in Europe and more frequent and widely scattered in Iran [184], Central and East Asia, and *O. moravicum* a very rare montane species both in Central Europe and East Asia. Finally, *Lewinskya vladikavkanum* is exceedingly rare in the Caucasus in Eastern Europe and in the Pontic Mountains in Turkey and appearing in Central Siberia and in China. In contrast, *L. iwatsukii* has a very wide geographical distribution, having a continuous range in Arctic and Subarctic Siberia and appearing in the mountains of Central Siberia and the southern part of the Russian Far East and extending to the Himalayan region of India, Nepal and Kashmir and to central and south-western China. In Europe it is known only from the Caucasus [145].

**Asian Temperate and Warm Temperate element**

Although Asia is a very vast continent, the Asian element does not constitute a significant group in China’s orthotrichalean mosses. It is represented by nine taxa (eight species and one variety) which occur in the temperate and warm temperate (subtropical) zones of Central and East Asia. They represent three distinct distribution patterns which are considered here as sub-elements.

*Sino–Central Asian sub-element*—It is represented by two species, which has hitherto been recorded only in the mountains of Central Asia and in China. It is likely that as exploration progresses, species so far known only from China will be discovered in Central Asian countries and vice versa. It is well confirmed by the history of the two species which currently represent this distribution pattern. *Orthotrichum pamiricum* was recently described as a new species from the Pamir of Tajikistan [185] and subsequently recorded in Kazakhstan [97] and Xinjiang Province in China [63]. On the other hand, *O. revolutum* was considered to be China’s endemic, but recently it was found in the Alay Range in the Pamir of Kyrgystan [186].

Orthotrichum pamiricum
O. revolutum

*Sino–Himalayan sub-element*—In a strict sense, this sub-element is defined as comprising taxa occurring in the Himalayan region (India, Kashmir, Nepal, Bhutan) and coterminous provinces of Yunnan and Sichuan in China [170]. However, herein the concept of this sub-element is expanded and species scattered in the adjacent provinces of Western, Central and East China in the temperate and subtropical zones are included. This sub-element consists of four taxa (three species and one variety).

Lewinskya hookeri
L. hookeri var. granulata
Orthotrichum crispifolium
O. griffithii

*Sino–Japanese sub-element*—This sub-element contains three species which occur at disjunct stations in Japan and in temperate and warm temperate regions of Central and East China but are lacking from the Himalayan region.

Orthotrichum consobrinum
O. erubescens
O. ibukiense

**North Amphipacific Temperate element**

This is a unique and exceptionally rare distribution pattern which is represented only by *Orthotrichum hallii*. It is similar to the well known disjunction in the occurrence of plants between Pacific North America and Pacific East Asia [161,162,187] but the essential difference is that *O. hallii* has a distinct centre of occurrence in Central Asia, including Xinjiang and Qinghai province in China [54], Kazakhstan [188] and the Altai in Russia [189]. Because the chain of the Altai Mountains appears to be an ancient refugium, one cannot reject the hypothesis of the Asiatic origin of this species and its subsequent migration eastwards to the Pacific coast of north-eastern Asia and north-western North America [190]. A somewhat similar geographical range is also shown by *Gollania turgens* (Müll.Hal.) Ando which occurs in Alaska, Sichuan and in the Sayan in Central Siberia [191].

**Eastern Asian–Eastern North American Temperate element**

This type of distribution is exhibited by species that are restricted to eastern North America and absent in western North America west of the Great Plains and in eastern Asia. Iwatsuki [192] listed 28 species of moss showing this disjunction, including *Leratia exigua* which is the only representative of this distribution pattern amongst China’s orthotrichalean mosses.

**Asian–Australasian element**

*Lewinskya graphiomitria* is an intriguing example of a remote intercontinental disjunction among the bryophytes. The species has hitherto been considered endemic to New Zealand and recently was found in Guizhou, Jiangxi and Taiwan Provinces, for the first time in Asia and in the Northern Hemisphere [66]. Only five species of moss share this rare distribution pattern, for example *Anacamptodon fortunei* Mitt. [193] and *Eccremidium minutum* (Mitt.) I.G. Stone and G.A.M. Scott [194].

**Bipolar element**

Bipolar element comprises taxa that occur in polar and cool-temperate regions of both hemispheres but are absent from the tropical lowlands and with or without intermediate occurrences at high elevations in the tropical regions. The latter feature is the basis for recognition of two sub-elements within this distribution pattern. Bipolar species are very rare among orthotrichalean mosses and this element is represented only by the two following species.

*Strict bipolar sub-element*—This includes *Orthotrichum cupulatum*, a pantemperate Holarctic species in the Northern Hemisphere which is also recorded from southern South America, south-eastern Australia and New Zealand in the Southern Hemisphere.

*Transitional bipolar sub-element*—The only species of this sub-element is *Lewinskya rupestris* which is a panholarctic disjunct oreophyte occurring from the Subarctic to the meridional zone in the southern part of the Holarctic. In the Southern Hemisphere it occurs in southern South America and Tierra del Fuego and extends to the northern maritime Antarctic [195], in Îles Kerguelen in the Subantarctic and in south-eastern Australia, Tasmania and the South Island of New Zealand [159].

## Figures and Tables

**Table 2:** Distribution of the taxa in the Chinese provinces. ○—literature data, ●—specimen-based records.

Taxa/Provinces	A	C	F	G	Gd	Gh	Gx	Ha	Hb	He	Hl	Hn	Hu	I	Jl	Js	Jx	L	N	Q	Sa	Sd	Sh	Si	Sx	Ta	Xi	Xz	Y	Z
***Florschuetziella scaberrima***																														
***Leratia exigua***	●	○	●			●							●			○	●				●			●					●	●
***Lewinskya affinis var. affinis***	●	○				●					○			●	●			○	○			●		●	○		●			●
***L. affinis var. bohemica***																											●			
***L. dasymitria***				○		●			●						●					●	○			●	●		●	●	●	
***L. erosa***				●		○			●												○			●						
***L. graphiomitria***						●											●									●				
***L. hookeri var. granulata***				○																	○			●				○	●	
***L. hookeri var. hookeri***		○		●		○														○	●			●			●	●	●	
***L. iwatsukii***						○														●				○			●	○	●	
***L. leiolecythis***				○					○					○	○		○			○	●			○	○		○	○	○	
***L. pulchra***						○														○				●						
***L. rupestris***													○								○			●			●	○	○	
***L. sordida***		○							●		●			●	●				○		○			○			●		○	○
***L. speciosa***		●		●					●	●	●		●	●	●			○		●	●			●	●	●	●	●	●	○
***L. striata***				●					●		○			○	●		●	○	○		●			●	●		●	●	○	
***L. taiwanensis***						○																				●		○		
***L. vladikavkana***																				●	●			●	●		●	●	●	
***Nyholmiella obtusifolia***				●						●	●			●	●		○	○	○	●	●	●		●			●		●	
***Orthotrichum alpestre***				●																							●	○		
***O. anomalum***			○			●				●	●			●			●		○	●	○	○		●	●		●	●	●	
***O. callistomum***				●		○														○	●			●	○	○		●	●	
***O. consobrinum***	●			○		●	○		○				●			●	○				●		●						●	●
***O. crenulatum***														●													●			
***O. crispifolium***	●					●			●				●							●	●			●		●			●	●
***O. cupulatum***																	●			○							●			
***O. erubecens***	○	○		○		○							○			○	●			○	○			○	○			○	○	●
***O. griffithii***	●	●				●				●							●			●	●			●					●	●
***O. hallii***																				●							○			
***O. ibukiense***				○						○														●				○	○	
***O. jetteae***				○		○			●				○											○						
***O. laxum***																				●								○		
***O. moravicum***																					●						●			
***O. notabile***																								●						
***O. pallens***						●								○						○					○		●			
***O. pamiricum***																											●			
***O. pellucidum***																											●			
***O. pumilum***		○								○				●						●	●						●			●
***O. revolutum***				○						●											●						●			
***O. rogeri***						●														●										
***O. scanicum***				●																	●						●			
***O. schimperi***																											●			
***O. schofieldii***	○			●																●				○						
***O. sinuosum***																					●									
***O. stramineum***	●			○																○				○			●		●	●
***O. subpumilum***	●	○	○										○			○	○			○				○					○	●
***O. urnigerum***															●									●			●			
***O. vermiferum***				○																●	○			○				○		
**Total number of taxa**	9	9	3	19	0	18	1	0	9	7	6	0	8	10	8	4	11	4	5	23	24	3	1	28	10	5	27	17	21	11

**Table 3:** Conspectus of phytogeographical elements of Chinese orthotrichalean mosses.

Category		Number of Taxa	Percentage of the Number of Taxa
**Endemic**		**13**	**27.0**
**Panholarctic**		**14**	**29.1**
**Eurasian**		**7**	**14.6**
**Asian**		**9**	**18.8**
	Sino-Central Asian	2	4.2
	Sino-Himalayan	4	8.4
	Sino-Japanese	3	6.2
**North Amphipacific**		**1**	**2.1**
**Eastern Asian Eastern North American**		**1**	**2.1**
**Asian Australasian**		**1**	**2.1**
**Bipolar**		**2**	**4.2**
	Strict	1	2.1
	Intermediate	1	2.1
**Total**		**48**	**100.0%**

## Data Availability

All authors agree with MDPI Research Data Policies.

## Supplementary Materials

The following are available online at https://www.mdpi.com/2223-7747/10/3/499/s1, **S1**: A list of herbarium specimens examined.
