# Supplementary material for: A Synopsis of Orthotrichum s. lato (Bryophyta, Orthotrichaceae) in China, with Distribution Maps and a Key to Determination
Source: Plants (Basel). 2021 Mar 8;10(3):499. doi: 10.3390/plants10030499 (PMC7999045; doi:10.3390/plants10030499)
Supplement: Supplementary file 1 [file plants-10-00499-s001.pdf]

S1: A list of herbarium specimens examined

A list of herbarium specimens examined is presented as the supplementary materials. See below for an explanation of the Chinese provinces abbreviations and acronyms of herbaria.

Provinces and autonomous regions of China: **A** – Anhui, **C** – Chongqing, **F** – Fujian, **G** – Gansu, **Gd** – Guangdong, **Gh** – Guizhou, **Gx** – Guangxi, **Ha** – Hainan, **Hb** – Hubei, **He** – Hebei, **Hl** – Heilongjiang, **Hn** – Henan, **Hu** – Hunan, **I** – Inner Mongolia, **Jl** – Jilin, **Js** – Jiangsu, **Jx** – Jiangxi, **L** – Liaoning, **N** – Ningxia, **Q** – Qinghai, **Sa** – Shaanxi, **Sd** – Shandong, **Sh** – Shanghai, **Si** – Sichuan, **Sx** – Shanxi, **Ta** – Taiwan, **Xi** – Xinjiang, **Xz** – Xizang, **Y** – Yunnan, **Z** – Zhejiang.

Acronyms of the herbaria according to *Index herbariorum* but for some Chinese herbaria they are unauthorised and introduced only for the purposes of the present article - they are distinguished by not using bold letters: **C** – University of Copenhagen, Denmark; **E** – Royal Botanic Garden Edinburgh, Scotland, U.K; GuZU – Guizhou University, China; **H** – University of Helsinky, Finland; **HSNU** – Herbaria of East China Normal University, China; HuBU – Hubei University, China; **IFP** – Institute of Applied Ecology, Academia Sinica, Shenyang, China; InMU – Inner Mongolia University, China; **KRAM** – Herbarium of the W. Szafer Institute of Botany, Polish Academy of Sciences, Krakow, Poland; **KUN** – Herbarium of Kunming Institute of Botany, the Chinese Academy of Sciences, China; **OSTR** – University of Ostrava, Czech Republic; **PC** – Muséum National d´Historie Naturelle, Paris, France; **PE** – Institut of Botany, Chinese Academy of Sience, Beijing, China; ShM – Shanghai Museum, China; ShU – Shanghai Normal University, China; **W** – Naturhistorisches Museum, Wien, Austria; **XJU** – Xinjiang University, China.

Leratia exigua (Sull.) Goffinet

Herbarium specimens examined: **A** (*OSTR* B3492, B3493; *ShM* 12038, 12047), **F** (*PE* 00667481, 00667508), **Gh** (*GuZU* DJ20160524090, DJ20160602020, F1040h; *OSTR* B2586, B2587, B2591, B2594, B2595, B2597, B2599, B2602, B2605), **Hu** (*KUN* B0010619), **Jx** (*PE* 00353696, 00353697A, 00353698A, 00353699A, 00353699B, 00353700A, 01077019; *ShM* 012109, 012111), **Sa** (*PE* 01090290), **Si** (*OSTR* B3494, B3668; *PC* 0878902; *PE* 00353758; *E* 04005410), **Y** (*KUN* B0010596, B0010597; *E* 00266289, 00266292, 00653850, 00670161), **Z** (*OSTR* B3491; *ShM* 12172).

Lewinskya affinis (Brid.) F. Lara, Garilleti & Goffinet **var. *affinis***

Herbarium specimens examined: **A** (*ShM* 12038, 12211, 12224; *ShU* 20160507017), **Gh** (*GuZU* DJ20160526032, DJ20160602019, DJ20160603012; *OSTR* B2591), **I** (*IFP* 0011597), **Jl** (*IFP* 002357; *PE* 00645488; *ShM* 12262, 12266), **Sd** (*IFP* 002356), **Si** (*IFP* 002349), **Xi** (*XJU* 196, 1526, 14914, 15139, 15269, 16324, 18611, 18670, 20759, 20790, 22972, 26662, 26783), **Z** (*ShU* 2011052402; *ShM* 12168).

Lewinskya affinis* var. *bohemica (Plášek & Sawicki) Plášek

Herbarium specimen examined: **Xi** (XJU 26783).

Lewinskya dasymitria (Lewinsky) F. Lara, Garilleti & Goffinet

Herbarium specimens examined: **Gh** (*OSTR* B2599, B2600, B2601), **Hb** (*IFP* 200973, 201043, 201063,), **Jl** (*IFP* 1157, 002355, 002360), **Q** (*PE* 00353685; *E* 27196a, 27196b, 00049025, 00151567) **Si** (*E* 39745, 39868, 39923, 40052, 40187; *GuZU* DG20120824040; *H* 45071, 46339, 91084; *KRAM* B-253655, B-253656, B-253657, B-253658, B-253659, B-253660, B-253661, B-253662, B-253663; *IFP* 10140; *KUN* B0010588, 0878642, 0878900; *PE* 01754596; *ShU* 90480, 201201, 201202, 201203, 2012082501, 2012082502, 2012082503, 2012082505, 2012082506, 2012082507, 2012082508, 2012082513; *XJU* 14539), **Sx** (*PE* 00648105), **Xi** (*IFP* 200293, 200353, *XJU* 11034, 13626, 14996, 15503, 15675, 16349, 23710, 26500, 26679), **Xz** (*PE* 01091968), **Y** (*E* 18562, 18719a, 23575, 23625a, 23690a, 23866a, 23945, 00151566, 00151568, 00151569, 00151571, 00151573, 00151574).

Lewinskya erosa (Lewinsky) F. Lara, Garilleti & Goffinet

Herbarium specimens examined: **G** (*PE* 01414559), **Hb** (*IFP* 200941, 201016), **Si** (*H* 91084; *PE* 01754596).

Lewinskya graphiomitria (Müll. Hal. *ex* Beckett) F. Lara, Garilleti & Goffinet

Herbarium specimens examined: **Gh** (*GuZU* DJ20160523159, DJ20160602019, DJ20160603012,), **Jx** (*ShM*

16651), **Ta** (*ShU* 20120915462, 20120915469, 20120915471).

Lewinskya hookeri* var. *granulata (Lewinsky) F. Lara, Garilleti & Goffinet
Herbarium specimens examined: **Si** (*OSTR* B1516), **Y** (*E* 18523, 18547, 18709, 18976, 23575, 23584, 23625a, 23651, 23690a, 23693, 23718, 23787, 23912, 23945, 24086, 24461, 00151560, 00151561, 00151562, 00151563, 00151564, 00151565).

Lewinskya hookeri (Wilson *ex* Mitt.) F. Lara, Garilleti & Goffinet var. ***hookeri***
Herbarium specimens examined: **G** (*ShU* 20060786-1), **Sa** (*ShU* 20160512032), **Si** (*E* 39718, 39726, 39746, 39790, 39859, 39957, 40017; *HSNU* 90478, 201204; *IFP* 19356, 19570, 19614, 19690, 19697, 19810, 19843, 20211, 20872; *KRAM* B-253619, B-253620, B-253621, B-253625, B-253626, B-253627, B-253628, B-253629, B-253630, B-253631, B-253632, B-253633, B-253634, B-253635, B-253636, B-253637, B-253638, B-253639, B-253640, B-253641, B-253642, B-253644, B-253655, B-253656, B-253657, B-253658, B-253659; *KUN* B0010589, 0855629, 0878429; *OSTR* B3663, B3664, B3666, B3667, B3669, B3691, B3692, B3693, B3694, B3695, B3696, B3697, B3698, B3699, B3700, B3701, B3702, B3703, B3704, B3705, B3706, B3707, B3708, B3709, B3710, B3711, B3712, B3713, B3714, B3715, B3716, B3717; *PE* 00353686, 00656560; *ShM* 2388, 5739; *ShU* 90288, 201202, 445079, 20120801, 20120802, 20120803, 20120804, 2012082502, 2012082508, 2012082512, 2012082513; *XJU* 14539), **Xi** (*PE* 1375, 5592; *XJU* 00095, 297, 00998, 5974, 8119, 12825, 12880, 13059, 13101, 13137, 13212, 13247, 13680, 13711, 13760, 13799, 13861, 13974, 14042, 14588, 14954, 15310, 15490, 15504, 15558, 15571, 15623, 16014, 16199, 16348, 16350, 16352, 17198, 17353, 18050, 18059, 18208, 18928, 19097, 20243, 20909, 23461, 23675, 23682, 24079, 24154, 24196, 24204, 24920, 24925, 24934, 25801, 25814, 25820, 26020, 26035, 26130, 26303, 26557, 26574, 27949, 28216, 28283, 28627, 811023590), **Xz** (*PE* 08483, 01758843; *KUN* 0878369), **Y** (*E* 18812, 18878, 19273, 23622, 23726, 23828, 24136, 24184, 24533; *H* 42031, 42053, 43086; *IFP* 002372; *KUN* B001058, B001574, B0010572, B0010577, B0010580, B0010587, B0010613, B0013626, 0878594, 0878896, 0879259, 0898056; *PE* 039102, 00353739; *ShU* G1007160022; *W* 3336, 3548, 4195, 6510, 6773).

Lewinskya iwatsukii (Ignatov) F. Lara, Garilleti & Goffinet
Herbarium specimens examined: **Q** (*E* 00049004; *PE* 00353745), **Xi** (*IFP* 420; *PE* 1413, 12811; *XJU* 5953, 12811, 12913, 14073, 14215, 15304, 18075, 18465, 26500, 28066), **Y** (*KUN* B0010584).

Lewinskya leiolecythis (Müll. Hal.) F. Lara, Garilleti & Goffinet
Herbarium specimens examined: **Sa** (*H* 2170)

Lewinskya pulchra (Lewinsky) F. Lara, Garilleti & Goffinet
Herbarium specimens examined: **Si** (*H* 45341, 46206; *IFP* 987; *KUN* 0878900; *PE* 00645497; *ShM* 985)

Lewinskya rupestris (Schwägr.) F. Lara, Garilleti & Goffinet
Herbarium specimens examined: **Si** (*E* 39692, 39818; OSTR B3663, B3665, B3666, B3667), **Xi** (*E* 01421490; *PE* 1419, 01747972; *XJU* K110, 346, 2846, 5552, 9022, 12808, 12812, 12907, 13783, 13832, 14215, 14913, 14966, 15203, 15270, 15307, 15313, 15319, 15321, 15326, 15331, 15643, 15644, 16834, 16898, 16901, 17353, 17751, 17755, 17770, 17787, 17805, 18597, 20230, 20886, 20972, 21331, 21614, 22943, 22957, 22962, 23152, 23301, 23347, 23356, 23366, 24079,24167, 24762, 25042, 25715, 26161, 26290, 26303, 26423, 26464, 26475, 26496, 26498, 26506, 26528, 26627, 26681, 26688, 26689, 26693, 26772, 27644, 27743, 27804, 27810, 27820, 27838, 27845, 27881, 27892, 27911, 27920, 27921, 27944, 27953, 28012, 28055, 28093, 28099, 28135, 28164, 28179, 28287, 28299, 28372, 28373, 28406, 28420, 28453, 28499, 28543, 28592, 240022).

Lewinskya sordida (Sull. & Lesq.) F. Lara, Garilleti & Goffinet
Herbarium specimens examined: **Hb** (*IFP* 200975), **Hl** (*IFP* 0008952, 0015662, 0015736), **I** (*IFP* 8776, 002344, 002362), **Jl** (*IFP* 000980, 002353, 002360, 002361, 36735a), **Xi** (*XJU* 10727, 24955).

Lewinskya speciosa (Nees) F. Lara, Garilleti & Goffinet
Herbarium specimens examined: **C** (*IFP* 5492), **G** (*KUN* B0010575; *PE* 01086817), **Hb** (*IFP* 200974, 200975), **He** (*HuBU* 98114, 971810, 971910-6), **Hl** (*IFP* 006169), **Hu** (*KUN* B0010615), **I** (*IFP* 0010609), **Jl** (*IFP* 002358, 002360, 002363, 18544; *PE* 01076829; *ShM* 09757, 10033, 10346), **Q** (*E* 26872, 27080, 27163b,

27196a, 27254, 27256c, 27294, 00049003, 00049007, 00049008, 00049012, 00049013, 00049016, 00049017, 00049018, 00049019, 00049020, 00049022, 00049023, 00049026, 00049027, 00049028), **Sa** (*OSTR* B3558, B3564; *ShU* 20160512021, 20160512026, 20160512041, 20160512045), **Si** (*H* 45071; *IFP* 000586; *OSTR* B3664, B3717; *ShU* 201201, 2012082401, 2012082405), **Sx** (*IFP* 12, 32), **Ta** (*ShU* 20120915425), **Xi** (*IFP* 447, 200210, 200328, 200374200413, 200494; *KUN* B0010592, B0010593; *PE* 1383, 2524, 15220, 138515139; *ShU* 201182601; *XJU* K76, K90, 2580, 2737, 2813, 04670, 5221, 5230, 5401, 5588, 9012, 9014, 9015, 9018, 10965, 11035, 11076, 11082, 11089, 11252, 12157, 12169, 12337, 12834, 12860, 13032, 13041, 13096, 13109, 13117, 13133, 13148, 13264, 13266, 13374, 13713, 13741, 13826, 13836, 13591, 13674, 13783, 14019, 14073, 14172, 14588, 14913, 15067, 15200, 15209, 15212, 15230, 15239, 15315, 15318, 15367, 15520, 15551, 15770, 15736, 16233, 16324, 16336, 16339, 16340, 16359, 16508, 16692, 16901, 17160, 17197, 17262, 17414, 17835, 17912, 17918, 17920, 17996, 18033, 18219, 18519, 18627, 18675, 19349, 19394, 19836, 20119, 20767, 20821, 20831, 21028, 21313, 21321, 21396, 21398, 21408, 21409, 21995, 22115, 22199, 22596, 22984, 23010, 23341, 23344, 23424, 23630, 23706, 23846, 23849, 23958, 24104, 24105, 24126, 24154, 24196, 24717, 25003, 25036, 25271, 25280, 26102, 26124, 26274, 26374, 27168, 27590, 27616, 27609, 28012, 28076, 28117, 28136, 28287, 28321, 28469, 28648, 29239, 29241) **Xz** (*IFP* 200206; *KUN* B0025815), **Y** (*IFP* 002367; *KUN* B0010591).

Lewinskya striata (Hedw.) F. Lara, Garilleti & Goffinet

Herbarium specimens examined: **G** (*PE* 01414559), **Hb** (*IFP* 201043), **Jl** (*IFP* 002359), **Jx** (*ShM* 016667), **Sa** (*OSTR* B3558, B3566; *ShU* 20160512031, 20160512041), **Sx** (*IFP* 19157), **Si** (*IFP* 003417; *KRAM* B-25369, B-253672, B-253610, B-253611, B-253692, B-253693; *OSTR* B3664; *ShU* 201202, 201203, 201204, 20120803, 20120804, 20120805; *XJU* 14539), **Xi** (*XJU* 24217, 25271, 25280), **Xz** (*IFP* 18612).

Lewinskya taiwanensis (Lewinsky) F. Lara, Garilleti & Goffinet

Herbarium specimens examined: **Ta** (*OSTR* B3999)

Lewinskya vladikavkana (Venturi) F. Lara, Garilleti & Goffinet

Herbarium specimens examined: **Q** (*E* 27080, 27196b, 00049012, 00049023), **Sa** (*PE* 00353687; *ShM* 5422), **Si** (*GuZU* DG20120824040, DG20120824052; *IFP* 982, 1955, 19857, 077933; *KRAM* B-253632, B-253633, B-253645, B-253646, B-253647, B-253662, B-253663, B-253664, B-253665, B-253699; *KUN* B0010571; *OSTR* B3669; *PE* 5828, 01745291, 00353686; *ShU* 2012082401, 2012082405, 2012082503, 2012082505, 2012082506), **Sx** (*PE* 645490), **Xi** (*PE* 1415; *XJU* 15309, 23010, 24105, 24212, 24217), **Xz** (*IFP* 17777), **Y** (*KUN* B0010577, B0010578, B0010579, 0878605; *OSTR* B3593, B3594, B3595, B3596, B3597, B3598, B3599, B3600, B3601, B3602, B3603, B3604, B3605, B3606, B3608, B3610, B3611, B3612, B36013, B3614, B3615, B3616; *PE* 00667215, 00667218; *ShU* G1007160024;).

Nyholmiella obtusifolia (Brid.) Holmen & E. Warncke

Herbarium specimens examined: **G** (*OSTR* B2580, B2581), **He** (*HuBU* 971815-Q, 971815-6, 971842, 971904,), **Hl** (*IFP* 002351), **I** (*IFP* 002344, 002348; *IM* 1944), **L** (*IFP* 38870), **Q** (*E* 27170-c, 27256-c, 00049014, 00049021, 00049030), **Sa** (*OSTR* B3548, B3549, B3550, B3551, B3552, B3553, B3554, B3555, B3557, B3559, B3563, B3567; *ShU* 20160512027), **Sd** (*IFP* 002356), **Si** (*E* 4009240092; *GuZU* DG20120823025; *IFP* 002349; *KRAM* B-253622, B-253623, B-253694, B-253695, B-253696, B-253697, B-253698, B-253699; *OSTR* B3662, B3663, B3664, B3670, B3671, B3672, B3673, B3674, B3675, B3676, B3677, B3678, B3679, B3680, B3681, B3682, B3683, B3684, B3685, B3686, B3687, B3688, B3689, B3690, B3691, B3692, B3693), **Xi** (*IFP* 200210, 200413; *XJU* 135, 263, 2813, 12845, 12869, 14454, 14549, 14550, 14583, 16057, 16059, 16212, 16214, 18519, 18530, 18547, 18670, 19303, 19798, 19830, 19835, 20761, 23301, 23319, 27590, 27601, 27691, 28300, 29425), **Y** (*E* 23615, 24476, 24514, 24518).

Orthotrichum alpestre Wilson

Herbarium specimens examined: **G** (*IFP* 8628), **Xi** (*PE* 1520; *XJU* 00740, 9941, 15326, 16898, 16901, 16902, 16965, 18587, 18595, 18600, 20135, 21027, 23436, 23551, 25712, 26285, 26459, 26506, 26507, 26627, 26696, 26761, 28177, 28350, 28480).

Orthotrichum anomalum Hedw.

Herbarium specimens examined: **Gh** (*GuZU* PX090501109, JS20150809008, JS20150809047, DJ20160526030, DJ20160526039), **He** (*HuBU* 88159, 97062, 98021, 98057, 98102, 98103-9, 98124-6, 98173, 98195, 971851, 971868; *IFP* 31654), **Hl** (*IFP* 002350), **I** (*IFP* 0011672, 0909655; *InMU* 0251; *PE* 00353569, 00353570), **Jx** (*ShM* 12109), **Q** (*E* 00049010, 00049011; *PE* 00353660), **Si** (*E* 39972), **Sx** (*IFP* 3), **Xi** (*HSNU*

07002; *IFP* 79, 419, 422, 428, 437, 02845, 03148, 03201, 8005, 8024, 200145, 200210, 200274, 200280, 200335, 200360, 200412, 200422, 200536; *KUN* B0010594, B0010595; *PE* 1505, 12769; *ShU* 201172301, 201172302, 211182301, 21182602, 21182604, 21182606; *XJU* 23, 38, 59, 64, 170, 00237, 294, 330, 344, 346, 00366, 00387, 00739, 00740, 2580, 2815, 2834, 3511, 5821, 6115, 9001, 9006, 9013, 9022, 9025, 9026, 9028, 9034, 9081, 09935, 09997, 11076, 12708, 12722, 12725, 12743, 12744, 12760, 12768, 12773, 12822, 12834, 12864, 12873, 12927, 12932, 12935, 12942, 12966, 12982, 13204, 13454, 13836, 13838, 14044, 14066, 14215, 14446, 14448, 14530, 14539, 14540, 14548, 14668, 14904, 15258, 15269, 15315, 15319, 16217, 16317, 16342, 16343, 16344, 16345, 16356, 16472, 16556, 16898, 16905, 16911, 17199, 17410, 17751, 17755, 17770, 17787, 17788, 17791, 17816, 18530, 18582, 18587, 18594, 18611, 18670, 18715, 18746, 18759, 18760, 18796, 19338, 19361, 19367, 19369, 19452, 19826, 19828, 19912, 19956, 20038, 20195, 20210, 20243, 20261, 20263, 20265, 20283, 20331, 20355, 20564, 20617, 20632, 20881, 20886, 20896, 20903, 20909, 20948, 20979, 21031, 21216, 21246, 21249, 21264, 21283, 21287, 21327, 21332, 21387, 21398, 21428, 21430, 21431, 21436, 21583, 21609, 21610, 21612, 21614, 21615, 21616, 21623, 21995, 22866, 22895, 22903, 22943, 22959, 23067, 23068, 23180, 23185, 23230, 23344, 23347, 23424, 23442, 23630, 23748, 23761, 23826, 23848, 23883, 23960, 24064, 24168B, 24210, 25170, 25171, 25211, 25268, 25941, 26135, 26149, 26219, 26290, 26296, 26303, 26311, 26377, 26459, 26496, 26528, 26681, 26688, 26679, 26781, 27152, 27157, 27168, 27173, 27181, 27186, 27187, 27600, 27609, 27621, 27635, 27644, 27703, 27745, 27751, 27810, 27813, 27822, 27911, 27942, 27659, 27970, 28037, 28135, 28167, 28168, 28174, 28179, 28284, 28287, 28300, 28327, 28350, 28372, 28420, 28480, 200228), **Xz** (*PE* 01089893), **Y** (*E* 18515, 18712, 24027, 24126).

Orthotrichum callistomum Fisch.-Oost. *ex* Bruch & Schimp.

Herbarium specimens examined: **G** (*PE* 01414559), **Sa** (*ShU* 20160512041), **Si** (*E* 39745, 40052, 40187; *H* 45071, 91084; *IFP* 3447, 077933; *KRAM* B-253627, B-253628, B-253629, B-253630, B-253634, B-253635, B-253636, B-253637, B-253638, B-253639, B-253642, B-253643, B-253644, B-253645, B-253646, B-253647, B-253648, B-253649, B-253650, B-253652, B-253653, B-253654, B-253657, B-253658, B-253659, B-253660, B-253661, B-253662, B-253663, B-253694, B-253695, B-253696; *KUN* 0878900; *OSTR* B3666, B3706, B3707, B3708; *PE* 01413425, 01754596; *ShU* 85, 201202, 20120801, 20120802, 20120803, 20120804, 2012082502, 2012082503, 2012082505; *XJU* 14539), **Xz** (*PE* 008183), **Y** (*E* 18655, 18719, 18976, 23625a, 23626, 23651, 23677, 23875, 23945, 24462, 24533, 41665, 00151564, 00151574, 00151575, 00151576, 00151579, 00151580, 185471664; *KUN* B0010573, B0012532; *OSTR* B3593, B3594, B3595, B3598, B3600, B3601, B3603, B3604; *PC* 0135109; *ShM* 6524, 7810; *ShU* 030674).

Orthotrichum consobrinum Cardot

Herbarium specimens examined: **A** (*OSTR* B3508, B3509, B3510, B3513, B3517, B3520, B3525, B3545; *ShM* 1728), **Gh** (*GuZU* MYH20130420032, DJ20160523159, DJ20160524081; *OSTR* B2607, B2590, B2605), **Hu** (*KUN* 0010617; *PC* 0167492; *ShM* 11538), **Js** (*ShM* 12039,12040), **Sa** (*ShU* 20160512021, 20160512026, 20160512027, 20160512041, 20160512043), **Sh** (*ShM* 12051), **Y** (*KUN* 0010585), **Z** (*GuZU* 07057, 07228, 08027; *OSTR* B3498, B3502, B3503, B3505, B3529, B35330, B3532, B3533, B3534, B3535, B3536, B3537, B3540, B3541, B3542, B3571, B3572, B3575, B3576, B3577, B3578, B3579, B3581, B3582, B3583, B3584, B3585, B3586, B3587, B3588, B3589, B3590, B3591, B3592; *ShM* 12162; *ShU* 20160503065, 20160503066, 20160503074, 20160503075, 20160504001, 20160504002, 20160504003, 20160504004, 20160504005, 20160504006).

Orthotrichum crenulatum Mitt.

Herbarium specimens examined: **I** (*IFP* 0011255, 0011332), **Xi** (*IFP* 0000096, 20150, 20411, 200164, 200413; *PE* 14511; *XJU* 36, 130, 11921, 14450, 14511, 15722, 15724, 16212, 16214, 16336, 16341, 17791, 18528, 18627, 19830, 21408, 21409, 21461, 23301, 23319, 24479, 24717, 25280, 27168).

Orthotrichum crispifolium Broth.

Herbarium specimens examined: **A** (*OSTR* B3497, B3506, B3507, B3508, B3510, B3511, B3512, B3514, B3515, B3519, B3521, B3522, B3523, B3524, B3526, B3527, B3543, B3544, B3545, B3560, B3574, B3619, B3620, B3621, B3622; *ShU* 20160506034, 20160506062, 20160506063, 20160507001, 20160507010, 20160507017, 20160507026), **Gh** (*GuZU* SN20140526008, DJ20160523159, DJ20160529047, DJ20160602019, DJ20160602020; *OSTR* B2588, B2589, B2590, B2595, B2599), **Hb** (*IFP* 201016), **Hu** (*KUN* B0010618), **Q** (*E* 27196a), **Sa** (*OSTR* B3546, B3547, B3548, B3549, B3550, B3551, B3552, B3553, B3554, B3555, B3556, B3557, B3561, B3562, B3563, B3564, B3565, B3566, B3568; *ShU* 20160510008, 20160512021, 20160512027, 20160512030, 20160512041, 20160512042, 20160512043, 20160512045), **Si** (*GuZU* DG20120823025; *IFP* 20500, 24462; *OSTR* B3718, B3719, B3720, B3721; *ShU* 20120806, 2012082404), **Ta** (*ShU* 20120915466, 20120916500, 20120916502), **Y** (*E* 18883, 23584, 41665,

00151586, 00151587; *KUN* 0010581, 0010582, 0010583; *OSTR* B3594, B3596, B3597, B3602, B3603, B3605, B3606, B3607, B3609, B3610, B3611, B3612, B3614, B3615, B3617, B3618; *PC* 0135103), **Z** (*OSTR* B3528, B3578, B3581, B3582; *PE* 00667479).

Orthotrichum cupulatum Brid.

Herbarium specimens examined: **Jx** (*ShM* 9987), **Xi** (*IFP* 420c, 420d, 428; *PE* 492, 498, 1490, 1516, 1532; *XJU* 498, 00603, 3517, 12764, 12830, 15319,18582, 19828, 21264, 21297, 23753, 28053).

Orthotrichum erubescens Müll. Hal.

Herbarium specimens examined: **Hu** (*OSTR* B3998), **Jx** (*PE* 00353702), **Z** (*HSNU* 20001737; *OSTR* B2588, B2590).

Orthotrichum griffithii Mitt. *ex* Dixon

Herbarium specimens examined: **A** (*OSTR* B3516; *ShU* 20160506011, 20160506021, 20160506033, 20160506062, 20160507010), **C** (*GuZU* S20120403108), **Gh** (*GuZU* F1040h, MYH20130420032, K20141125086, JS20150808063, DJ20160523007, DJ20160526040; *OSTR* B2589, B2592, B2594, B2595, B2596, B2597, B2598, B2600, B2601, B2604, B2606,), **He** (*HuBU* 971821), **Jx** (*ShM* 10024,12132), **Q** (*E* 27080, 27196c, 00049025), **Sa** (*OSTR* B3559), **Si** (*IFP* 6816), **Y** (*E* 24518, 00266290, 00701765), **Z** (*HSNU* 20001735, 20001738, 20001739, 20001740; *OSTR* B3531; *ShM* 12162; *ShU* 20170426026, 20170426029).

Orthotrichum hallii Sull. & Lesq.

Herbarium specimen examined: **Q** (*E* 00049005).

Orthotrichum ibukiense Toyama

Herbarium specimen examined: **Si** (*OSTR* B3997).

Orthotrichum jetteae B. H. Allen

Herbarium specimen examined: **Hb** (*IFP* 200941).

Orthotrichum laxum Lewinsky

Herbarium specimen examined: **Q** (*E* 26961).

Orthotrichum moravicum Plášek & Sawicki

Herbarium specimens examined: **Sa** (*OSTR* B3557), **Xi** (*XJU* 12986, 27697).

Orthotrichum notabile Lewinsky

Herbarium specimen examined: **Si** (*OSTR* B3994).

Orthotrichum pallens Brid.

Herbarium specimens examined: **Gh** (*OSTR* 2593); **Xi** (*XJU* 135, 12986, 14441, 14517, 14573, 14585, 14588, 16344, 18521, 18618, 18620, 19303, 19835, 19852, 21583, 22023, 23344, 23706, 23855, 24555, 25003, 25271, 28486).

Orthotrichum pamiricum Plášek & Sawicki

Herbarium specimens examined: **Xi** (*IFP* 200164; *ShU* 201182601, 201182602, 201182603, 201182605; *XJU* 0244, 9015, 9018, 9026, 9034, 11076, 11921, 11252, 12147, 14583, 16057, 16059, 16212, 16233, 16337, 16339, 17912, 17918, 18430, 18532, 18534, 18535, 18543, 18547, 18553, 18576, 18609, 18670, 18675, 18715, 19798, 19835, 19836, 19852, 19862, 20119, 20150, 21405, 21480, 23777, 23846, 24304, 24406, 24416, 24455, 24462, 24473, 24493, 24640, 24652, 24738, 25506, 27330, 27590, 27601, 27616, 27691, 27751, 28300, 28592, 29425).

Orthotrichum pellucidum Lindb.

Herbarium specimen examined: **Xi** (*OSTR* B3991).

Orthotrichum pumilum Sw.

Herbarium specimens examined: **I** (*IFP* 0011401, 0011528), **Q** (*E* 27066b, 27162b, 27214b, 27256b, 00049013, 00049018, 00049026), **Sa** (*OSTR* B3549, B3567), **Xi** (*IFP* 200151, 200164, 200210, 200410; *PE* 14511; *ShU* 201182604; *XJU* 130, 00301, 14441, 14454, 14455, 14517, 14529, 18530, 18533, 18534, 19862, 20193, 20761, 20787, 20790, 22549, 23301, 23319, 23706, 24640, 25271, 27609, 27751, 28300), **Z** (*HSNU* 20001741).

Orthotrichum revolutum Müll. Hal.

Herbarium s*pecimens examined:* **He** (*HuBU* 20451), **Sa** (*HuBU* 2996026), **Xi** (*XJU* 2813, 20821).

Orthotrichum rogeri Brid.

Herbarium specimens examined: **Gh** (*GuZU* LP0310615), **Q** (*E* 27163c, 27248b).

Orthotrichum scanicum Grönvall

Herbarium specimens examined: **G** (*XJU* 54349), **Sa** (*OSTR* B3548, B3550, B3551, B3552, B3553, B3563, B3567, B3568), **Xi** (*XJU* 192, 00241, 294, 325, 455, 00998, 04670, 12834, 12986, 16340, 17199, 17262, 17996, 18547, 18560, 18582, 18587, 18741, 18746, 19836, 20763, 20767, 21216, 21405, 22023, 23531, 23699, 23958, 24126, 24640, 26149, 26528, 26693, 26783, 27168).

Orthotrichum schimperi Hammar

Herbarium specimens examined: **Xi** (*XJU* 12986, 22549).

Orthotrichum schofieldii (B. C. Tan & Y. Jia) B. H. Allen

Herbarium specimens examined: **G** (*XJU* 54349), **Q** (*E* 27196b, 00049012, 00049029, 000207020, 000207021).

Orthotrichum sinuosum Lewinsky

Herbarium specimen examined: **Sa** (*OSTR* B3984).

Orthotrichum stramineum Brid.

Herbarium specimens examined: **A** (*OSTR* B3498), **Xi** (*XJU* 26503), **Y** (*E* 24518), **Z** (*HSNU* 035182).

Orthotrichum subpumilum E. B. Bratram *ex* Lewinsky

Recent collections: **A** (*OSTR* B3516), **Z** (*OSTR* B3531).

Orthotrichum urnigerum Myrin

Herbarium specimens examined: **Jl** (*IFP* 7251), **Si** (*H* 45738), **Xi** (*XJU* 26358, 26759, 28406, 28666).

Orthotrichum vermiferum Lewinsky

Herbarium specimen examined: **Q** (*E* 27179a).
